# Evolutionary divergence of the plant elicitor peptides (Peps) and their receptors: interfamily incompatibility of perception but compatibility of downstream signalling

**DOI:** 10.1093/jxb/erv236

**Published:** 2015-05-22

**Authors:** Martina Lori, Marcel C. van Verk, Tim Hander, Hendrik Schatowitz, Dominik Klauser, Pascale Flury, Christoph A. Gehring, Thomas Boller, Sebastian Bartels

**Affiliations:** ^1^Zürich-Basel Plant Science Center, Department of Environmental Sciences – Botany, University of Basel, Hebelstrasse 1, CH-4056 Basel, Switzerland; ^2^Plant-Microbe Interactions, Department of Biology, Faculty of Science, Utrecht University, Padualaan 8, 3584 CH Utrecht, The Netherlands; ^3^Bioinformatics, Department of Biology, Faculty of Science, Utrecht University, Padualaan 8, 3584 CH Utrecht, The Netherlands; ^4^Division of Biological & Environmental Science & Engineering, 4700 King Abdullah University of Science and Technology, Thuwal 23955-6900, Kingdom of Saudi Arabia

**Keywords:** DAMP, Pep, PEPR, peptide evolution, PROPEP, PTI.

## Abstract

Plant elicitor peptides (Peps) are potent inducers of pattern-triggered immunity and amplify the immune response against diverse pathogens. Peps have been discovered and studied extensively in *Arabidopsis* and only recently orthologues in maize were also identified and characterized in more detail.

Here, the presence of PROPEPs, the Pep precursors, and PEPRs, the Pep receptors, was investigated within the plant kingdom. PROPEPs and PEPRs were identified in most sequenced species of the angiosperms. The conservation and compatibility of the Pep-PEPR-system was analysed by using plants of two distantly related dicot families, Brassicaceae and Solanaceae, and a representative family of monocot plants, the Poaceae. All three plant families contain important crop plants, including maize, rice, tomato, potato, and canola. Peps were not recognized by species outside of their plant family of origin, apparently because of a divergence of the Pep sequences. Three family-specific Pep motifs were defined and the integration of such a motif into the Pep sequence of an unrelated Pep enabled its perception. Transient transformation of *Nicotiana benthamiana* with the coding sequences of the AtPEPR1 and ZmPEPR1a led to the recognition of Pep peptides of Brassicaceae or Poaceae origin, respectively, and to the proper activation of downstream signalling. It was concluded that signalling machinery downstream of the PEPRs is highly conserved whereas the leucine-rich repeat domains of the PEPRs co-evolved with the Peps, leading to distinct motifs and, with it, interfamily incompatibility.

## Highlight

Plant elicitor peptides (Peps) co-evolved with their receptors, resulting in interfamily incompatibility of Pep recognition. In contrast, operation of defence pathways by Pep receptors is conserved within the flowering plants.

## Introduction

Plant immunity is triggered by the recognition of exogenous as well as endogenous elicitors. Microbe-associated molecular patterns are well-known representatives of the former whereas the latter are often classified as danger- or damage-associated molecular patterns (DAMPs) (Boller and Felix, 2009; Ferrari *et al.*, 2013; Albert, 2013). Plant elicitor peptides (Peps) are emerging as paradigms for DAMPs owing to their presence in dicot as well as monocot model plants and their supposed release upon damage (Huffaker *et al.*, 2011; Bartels *et al.*, 2013; Yamaguchi and Huffaker, 2011). In brief, Peps mature from larger precursor proteins called PROPEPs and are recognized by leucine-rich repeat (LRR) receptor-like kinases known as Pep receptors (PEPRs). Pre-treatment of *Arabidopsis* or *Zea mays* (maize) plants with Peps triggers defence responses and significantly improves their resistance against diverse pathogens, including bacteria and fungi, as well as herbivores (Huffaker *et al.*, 2006; Yamaguchi *et al.*, 2010; Huffaker *et al.*, 2011; Huffaker *et al.*, 2013; Liu *et al.*, 2013; Tintor *et al.*, 2013).

In *Arabidopsis*, eight PROPEPs (PROPEP1-8) and two PEPRs (PEPR1 and PEPR2) have been identified (Krol *et al.*, 2010; Yamaguchi *et al.*, 2010; Bartels *et al.*, 2013). Current models suggest a cleavage or processing of PROPEPs to produce Peps, which represent roughly the last 23 amino acids of the C-terminal part of the PROPEPs (Yamaguchi and Huffaker, 2011). Individual PROPEPs have been shown to localize to the cytoplasm or to be associated with the tonoplast, contributing to the assumption that Peps are released into the apoplast either actively as a response to danger signals or passively during damage and loss of cell integrity (Bartels *et al.*, 2013). Once in the apoplast they can reach PEPRs of adjacent cells and trigger and/or amplify immunity.

Little is known about Peps and PROPEPs and even less about PEPRs in other dicot plants. A small member of Peps from Solanaceae [*Solanum melongena* (aubergine) SmPep1, *Capsicum annuum* (pepper) CaPep1, and *S. tuberosum* (potato) StPep1] and Fabaceae [*Glycine max* (soybean) GmPep3, *Medicago truncatula* MtPep1, and *Arachis hypogaea* (peanut) AhPep1] was shown to induce the release of volatiles, a typical defence response against herbivore attack (Huffaker *et al.*, 2013). In addition a recent study reported the reduced expression of defence-related genes as well as a reduced resistance towards the necrotrophic fungus *Pythium dissotocum* in *Solanum lycopersicum* (tomato) plants upon silencing of a putative tomato *PROPEP* (Trivilin *et al.*, 2014).

In maize, two PROPEPs have been studied in more detail, ZmPROPEP1 and ZmPROPEP3 (Huffaker *et al.*, 2011; Huffaker *et al.*, 2013). The former is induced upon fungal infections whereas the latter is induced upon application of *Spodoptera exigua* oral secretions. Accordingly, treatments with ZmPep1 and ZmPep3 led to an upregulation of defence-related genes and improved resistance against fungal infections and herbivore feeding (Huffaker *et al.*, 2011; Huffaker *et al.*, 2013).

In *Arabidopsis*, an alanine-substitution approach has been used to identify the crucial amino acids for Pep perception by PEPRs (Pearce *et al.*, 2008). In this study a minimum core of the last 15 amino acids of AtPep1 [AtPep1(9–23)] showed a comparably similar activity as the unmodified AtPep1. Moreover, the exchange of serine^15^ or glycine^17^ for alanine as well as the deletion of the terminal asparagine^23^ produced a dramatic decrease in AtPep1 activity (Pearce *et al.*, 2008). Recently, the crystal structure of the AtPEPR1-LRR domain in complex with AtPep1 was released (Tang *et al.*, 2015). The authors reported that, in particular, the C-terminal 10 residues of AtPep1 interact closely with the AtPEPR1-LRR, and they include the previously described and conserved ser^15^, gly^17^, and asp^23^. In addition, modelling of the PEPR1-LRR/AtPep1/BAK1-LRR complex indicated that proline^19^ as well as glutamine^21^ and histidine^22^ are important for the PEPR1 BAK1 (BRI1-associated kinase1) interaction. This interaction has been shown before to be crucial for mounting full-strength defence responses upon AtPep1 perception (Roux *et al.*, 2011).

Despite the apparent common defence-amplifying action of PROPEPs from plant species as diverse as *Arabidopsis* and maize, their amino acid-based homology is very low (Huffaker *et al.*, 2011). Even among the PROPEPs from *Arabidopsis* there is only an overall amino acid sequence identity between 12% and 47% (Yamaguchi *et al.*, 2006). Moreover, already published Pep sequences show alterations in the conserved key amino acids, for example ZmPep1 has a C-terminal his^23^ instead of the asp^23^ whereas Peps of the Solanaceae show a gly^15^ instead of a ser^15^ (Huffaker *et al.*, 2013). In contrast, ZmPep3 is recognized by neither aubergine (Solanaceae) nor soybean (Fabaceae), despite the presence of ser^15^, gly^17^, and asp^23^ (Huffaker *et al.*, 2013).

Here, a comprehensive search for PROPEPs and PEPRs throughout the plant kingdom was performed, taking into account the many recently sequenced plant genomes. The elicitor-triggered release of ethylene was used as a robust and widespread read-out to investigate the interspecies recognition of known and newly identified Peps from the many dicot crop plants in the Brassicaceae and Solanaceae and the monocot crops in the Poaceae. Peps from one plant family are generally not perceived by plants belonging to another plant family despite the presence of PEPRs. Individual sequence alignment of all tested Peps from one family revealed family-specific Pep motifs. Inclusion of family-specific motifs into the sequence of incompatible Peps enabled their recognition. Further, functional PEPRs were cloned from tomato and maize. Transient expression of AtPEPR1 and ZmPEPR1a in *Nicotiana benthamiana* led to AtPep1 and ZmPep1 sensitivity indicating that, in contrast to Peps, PEPRs are interspecies compatible.

## Material and methods

### Plant material

*Arabidopsis thaliana* ‘Col-0’ and *Brassica rapa* plants were grown individually in small pots at 21°C and a 10h photoperiod for 4–5 weeks. Plants of the species *S. lycopersicum*, *N. benthamiana*, *Z. mays*, and *Lolium perenne* were grown as single plants per pot at 24°C and a 16h photoperiod for 3–5 weeks.

### Peptides

Peptides were obtained from Selleckchem (Houston, TX, USA) and dissolved in a solution containing 1mg/mL bovine serum albumin and 0.1M NaCl to reach stock concentrations of 100 µM. Further dilutions were done in double-distilled H_2_O. The list of peptides and their sequences can be found in Supplementary Table S1.

### Bioinformatics

Novel PEPR sequences were identified using NCBI blastp as well as tblastn on phytozome.com using the AtPEPR1 and AtPEPR2 sequences. For the identification of novel PROPEP sequences, all sequences from Bartels *et al.* (2013) and Huffaker *et al.* (2013) were aligned per plant family and used as input for an hmmsearch search (HMMER v1.9; (Finn *et al.*, 2011)) against the NR, RefSeq, and UniProtKB databases with standard settings. Identified sequences were manually curated for the presence of a Pep motif at the C-terminal end of protein. Newly identified PROPEPs were used as additional input for a new hmmsearch. All identified PEPRs and PROPEPs are listed in Supplementary Table S2.

Identification of the kinase and LRR domain within the PEPR sequences was done by scanning for full Pfam domains (Finn *et al.*, 2014) with default settings using CLC Main Workbench 6.7.1 (CLC bio, Aarhus, Denmark). For building the trees and identity graphs, all sequences were first aligned using CLC Main Workbench 6.7.1 (CLC bio) and subsequently identities were called or the trees built using neighbour-joining with 1000 bootstraps.

Visualization of Pep consensus sequences was done using WebLogo 2.8.2 (http://weblogo.berkeley.edu/logo.cgi) (Crooks *et al.*, 2004).

### Ethylene measurement

For wild-type plants, 8–10 leaf discs (5mm diameter cork borer) or equal leaf squares (cut with scissors) were harvested from fully expanded leaves and placed into a 6mL glass vial containing 0.5mL of double-distilled H_2_O. In case of transiently transformed *N. benthamiana* leaves, discs were harvested from at least three independently transformed leaves, mixed, and distributed into the vials (three each). After a 16h incubation period in the growth chamber, elicitor peptides (1 μM final concentration) were added and vials were closed with air-tight rubber septa. Vials were incubated for 5h at room temperature before ethylene accumulating in the free air space was measured by gas chromatography (GC-14A Shimadzu).

### Cloning of ZmPEPR1a and SlPEPR1

Total RNA of *Z. mays* and *S. lycopersicum* was extracted from a 1:1 mix of root and leaf material of 3-week-old plants using Nucleospin RNA plant spin columns (Macherey-Nagel). Reverse transcription of mRNA into cDNA was performed using an AMV-RT enzyme kit (Promega) together with a 21 nucelotide oligo dT primer. The ZmPEPR1a coding sequence was amplified from *Z. mays* cDNA using forward primer (5′-GGGACAAGTTTGTAC AAAAAAGCAGGCTTGATGAAGCTGGTTTTCTGGCATTG GATTTTTCTATTCTTC-3′) and reverse primer (5′-GGGGACC ACTTTGTACAAGAAAGCTGGGTCCTGCCGGTAGGCGCT GCTGTTGGA TTGCGATCCTG-3′) in a PCR reaction with Phusion polymerase (New England Bio Labs) in GC reaction buffer and 3% DMSO for amplification of GC-rich targets to generate a 3429 base pair product. The SlPEPR1 coding sequence was amplified from *S. lycopersicum* cDNA using forward primer (5′-GGGACAAGTTTGT ACAAAAAAGCAGGCTTGATGAAGATAGCTGTTCATAATT TGATCTTTTTCTACTGC-3′) and reverse primer (5′-GGGGACCA CTTTGTACAAGAAAGCTGGGTCGTACTTGCTTCGTATAC TCGAACTTGACCTTGTTAATAG-3′) in a standard PCR reaction with Phusion polymerase to generate a 3372 base pair product. Correct PCR products were cloned into pDONR207, sequenced, and subcloned into pGWB517 using the Gateway cloning technique according to the manufacturer’s protocol (Invitrogen).

### Transient expression of PEPRs in *N. benthamiana*

*Agrobacterium tumefaciens* GV3101 strains harbouring pGWB517 plasmids with either the coding sequence of AtPEPR1, SlPEPR1, or ZmPEPR1a were grown for 24h in liquid YEB medium supplemented with appropriate antibiotics. Harvested cultures were re-suspended in a solution containing 10mM MES (pH 5.6) and 10mM MgCl_2_ to reach OD_600_ = 0.1, and syringe inﬁltrated into 3-week-old *N. benthamiana* leaves. Infiltrated leaf areas were harvested 24h after infiltration and used for the measurement of ethylene production upon peptide treatment as described above.

## Results

### Identification of PROPEP and PEPR homologues in multiple plant species within the angiosperms

**Fig. 1.**
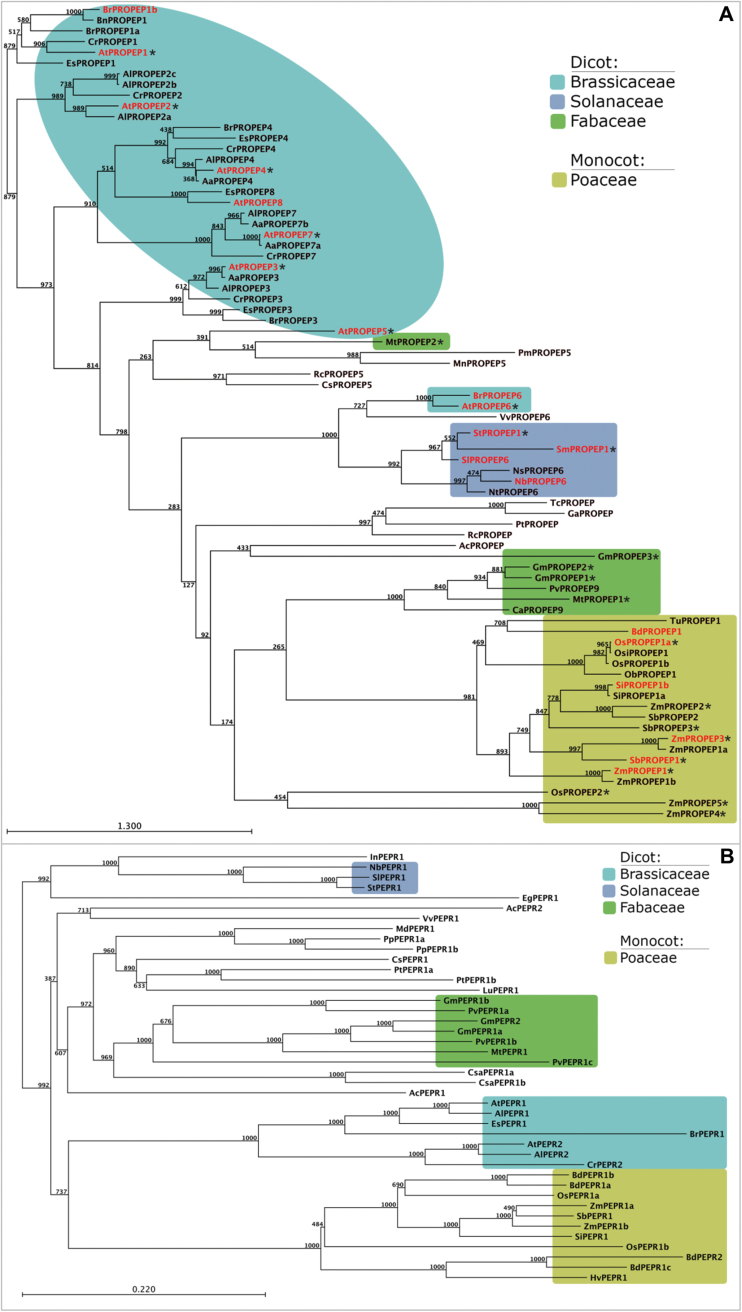
**Bootstrapped neighbour-joining tree of PROPEP and PEPR sequences.** (A) Full-length amino-acid sequences of published and novel HMMER identified PROPEP sequences were used to build a bootstrapped neighbour-joining tree. PROPEPs in red highlight PROPEPs of which the respective Pep was shown to be an active elicitor in this study. Asterisks mark PROPEPs of which the respective Pep was shown in previous studies to be an active elicitor. Major families are highlighted with colours according to the legend. Scale-bar: amino-acid substitutions per site. (B) Full-length amino-acid sequences of PEPR sequences were used to build a bootstrapped neighbour-joining tree. Major families are highlighted with colours according to the legend. Scale-bar: amino-acid substitutions per site.

The structure and function of the Pep-PEPR system has been studied mainly in the model plants *A. thaliana* and *Z. mays* (Huffaker *et al.*, 2006; Krol *et al.*, 2010; Yamaguchi *et al.*, 2010; Huffaker *et al.*, 2011; Bartels *et al.*, 2013). However, already in the earliest of these publications was the suggestion that PROPEPs might be present in a couple of plant species and not limited to *Arabidopsis* (Huffaker *et al.*, 2006). Here, an extensive sequence search in public databases using the few previously described PROPEP sequences as well as the sequences of the hitherto only known PEPRs, AtPEPR1 and AtPEPR2, identified a large number of novel PROPEPs and PEPRs (Supplementary Table S2). Figure 1 shows the phylogenetic trees of all PROPEPs (Fig. 1A) and PEPRs (Fig. 1B). PROPEPs form plant family–specific clusters, for example AtPROPEP1 clusters primarily with most Brassicaceae PROPEPs and not with PROPEP1 orthologues of distantly related plant species. A sequence comparison of all identified PROPEPs found an astonishingly small sequence identity between PROPEPs (Supplementary Table S3). For example the orthologues AtPROPEP1/ZmPROPEP1 and AtPROPEP3/ZmPROPEP3, which have been linked to fungal and herbivore resistance, respectively (Huffaker *et al.*, 2006; Huffaker *et al.*, 2011; Huffaker *et al.*, 2013; Liu *et al.*, 2013; Klauser *et al.*, 2015), show as little as 5.5% and 5.3% identical amino acids, respectively. In general, a large number of PROPEPs show less than 10% identical amino acids compared to other PROPEPs. Only within family-specific clusters and subclusters inside the Brassicaceae sequence identity ranged above 50% (Supplementary Table S3). It has been proposed that the PROPEP C-terminal end containing the Pep sequence is more strictly conserved (Huffaker *et al.*, 2006; Pearce *et al.*, 2008). However, a comparison of AtPep1 and ZmPep1, the first plant ortholog reported, revealed only a Pep-sequence identity of 20.8%; this is a typical value for the sequence conservation of Peps in general (Supplementary Table S4). It is slightly higher than the typical value for PROPEPs but still too low to support the idea that whole Pep sequences are strictly conserved over the whole structure. Notably, the sequence identity of Peps within a cluster, even as large as the one of the Poaceae Peps, ranged from 43.5% to 100% (Supplementary Table S4).

To date there are two exceptions to the apparent rule that PROPEPs form family-specific clusters: AtPROPEP5 and AtPROPEP6. Especially the latter seems to be closely related to the PROPEPs of the Solanaceae and groups within their family cluster. Therefore, it is proposed that the two already described PROPEPs, StPROPEP1 and SmPROPEP1, be reclassified as StPROPEP6 and SmPROPEP6, respectively.

In comparison to the limited number of PROPEPs in other plant families, there was a clear over-representation of PROPEPs from species belonging to the Brassicaceae. Provided that other plant genomes are as well annotated as the ones from *Arabidopsis* and its relatives, it seems that there was a recent multiplication of *PROPEP*s in the genome of a Brassicaceae ancestor and not in dicot species. A similar number of PROPEPs within one species has only been found in the monocot species maize (seven PROPEPs) and *Oriza sativa* Japonica (rice, three PROPEPs) (Fig. 1A and Supplementary Table S2).

Regarding PEPRs it seems that to date most species contain only one PEPR (Fig. 1B), although two have been characterized in *Arabidopsis* (Krol *et al.*, 2010; Yamaguchi *et al.*, 2010). Similar to PROPEPs, PEPR sequences formed family-specific clusters (Fig. 1B) with sequence identities ranging from 60% to 90% within a family cluster (Supplementary Table S5). Contrary to the low overall conservation of the PROPEPs, the overall level of conservation of the PEPRs was around 40% sequence identity higher.

So far no PROPEPs or PEPRs have been identified outside the angiosperms.

### Interfamily incompatibility of Peps

**Fig. 2.**
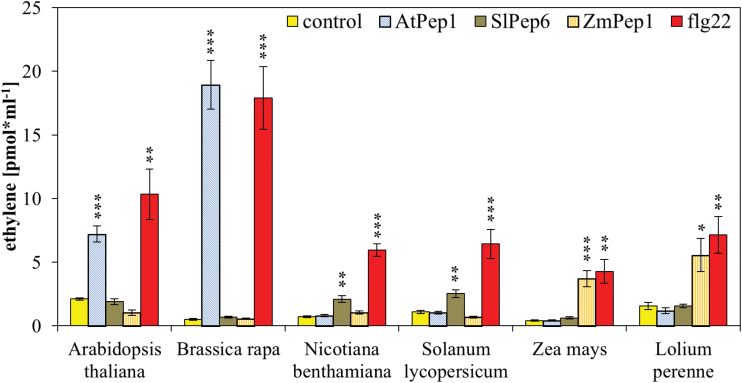
**Interfamily incompatibility of Peps.** Eight to ten leaf discs of indicated plant species were treated for 5h with 1 µM of the indicated elicitor peptides or without any peptide (control). Columns represent averages of detected ethylene values of five biological replicates. Error bars show the standard error of the mean. Asterisks indicate significant differences of the labelled column to the control based on t-test results (*P < 0.05; *P < 0.01; ***P < 0.001).

Given the aforementioned variability of the PROPEP as well as Pep sequences, the question arises, what is the structural basis of Pep perception and specificity. A first report in 2013 indicated that aubergine and soybean do not perceive Peps originating from species outside the Solanaceae and Fabaceae, respectively (Huffaker *et al.*, 2013). However, the authors used only these two species together with the monitoring of volatile production to characterize the perception of Peps and it is currently not certain if volatile emission is a typical response triggered by Pep binding to PEPRs. Here, the production of ethylene was used as a robust and reliable output. This has been used by multiple studies characterizing Pep responses, in conjunction with additional pattern-triggered immunity (PTI)-related responses like the production of reactive oxygen species or the phosphorylation of MAP kinases, to monitor the perception of Peps in a number of different species (Krol *et al.*, 2010; Roux *et al.*, 2011; Bartels *et al.*, 2013; Flury *et al.*, 2013). Two species from each of the distantly related plant families Brassicaceae (*A. thaliana* and *B. rapa*), Solanaceae (*S. lycopersicum* and *N. benthamiana*), and Poaceae (*Z. mays* and *L. perenne*) were chosen, together with a representative peptide (AtPep1, SlPep6, and ZmPep1, respectively), to determine interspecies and interfamily perception of Peps. As shown in Fig. 2, AtPep1 was only perceived by *Arabidopsis* and its close relative *B. rapa*, causing a highly significant release of ethylene absent in the more distantly related species. The same was true for the perception of SlPep6 and ZmPep1, which were only perceived by the species belonging to the same plant family (Fig. 2). Taken together, there seems to be an interfamily but not an interspecies incompatibility of Pep perception.

### Determination of novel family-specific Pep motifs

The molecular characteristics of Pep recognition have been exclusively studied in *Arabidopsis*. An alanine-substitution approach using AtPep1 in combination with monitoring the medium alkalinization response led to two major findings: (i) A minimum core of the last 15 amino acids of AtPep1 [AtPep1(9–23)] is sufficient to cause activity comparable to that of full-length AtPep1, whereas (ii) exchange of serine^15^ or glycine^17^ for alanine or deletion of the terminal asparagine^23^ almost completely abolishes elicitation of the alkalinization response (Pearce *et al.*, 2008). Thus it seems that the motif SxGxxxxxN, which is strictly conserved within all eight AtPeps, is critical for Pep activity.

**Fig. 3.**
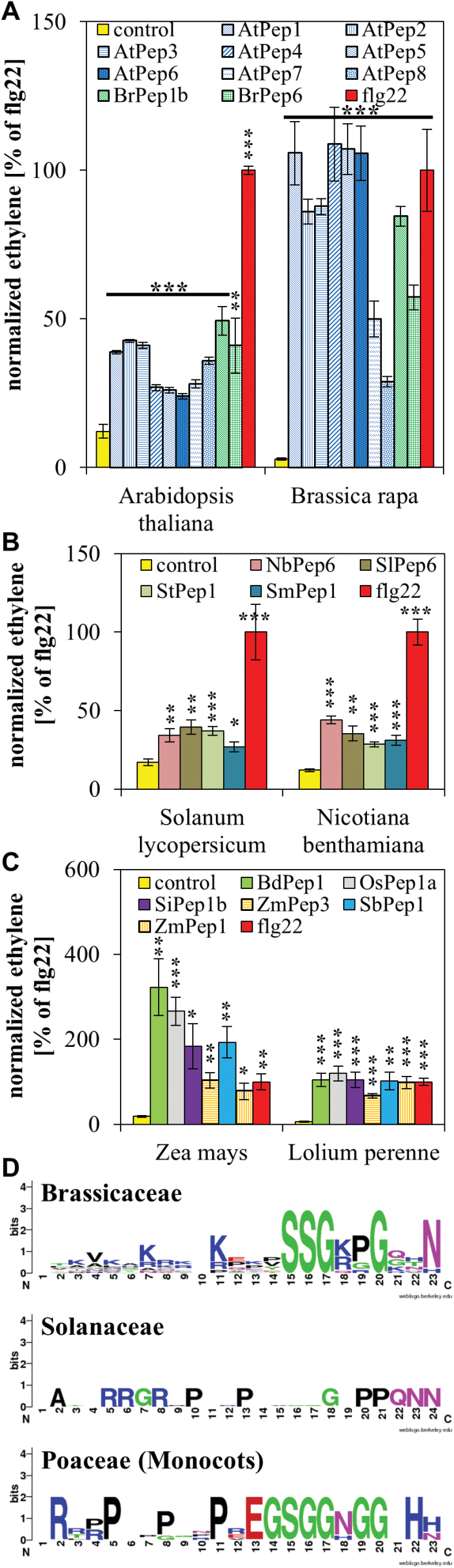
**Identification of family-specific Pep motifs.** (A–C) Ten leaf discs of indicated plant species were treated for 5h with 1 µM of the indicated elicitor peptides or without any peptide (control). Columns represent averages of detected ethylene values of five biological replicates normalized to the ethylene response triggered by flg22 (set to 100%). Error bars show the normalized standard error of the mean. Asterisks indicate significant differences of the labelled column to the control based on t-test results (*P < 0.05; *P < 0.01; ***P < 0.001). (D) Depiction of the consensus sequences of aligned Brassicaceae-, Solanaceae-, and Poaceae-specific Pep sequences (from A–C) using the WebLogo tool (Crooks *et al.*, 2004).

The peptides SlPep6 and ZmPep1 used in Fig. 2 do not conform to this rule. SlPep6 contains a glycine at position 15 instead of a serine and ZmPep1 shows a histidine at the terminal position 23 instead of an asparagine. This might explain why they were not recognized by the Brassicaceae. In order to identify plant family-specific motifs, a larger number of family-specific peptides were tested and consensus sequences derived. Fig. 3A shows the recognition of all eight AtPeps and two BrPeps from *B. rapa* by *Arabidopsis* and *B. rapa*. In Fig. 3B, a similar experiment is shown using four peptides from Solanaceae together with *S. lycopersicum* and *N. benthamiana*. Fig. 3C shows six peptides from Poaceae tested using *Z. mays* and *L. perenne*. Consistently, the collection of family-specific peptides triggered a significant induction of ethylene production, indicating that these peptides were perceived by the respective species. This indicates that the Peps derived from the newly identified PROPEPs are indeed active Peps and that all peptides related to a plant family are recognized by (at least two) species from this plant family. Given these findings, the Pep sequences were used to build a weblogo for the visualization of the consensus sequence of each peptide group (Fig. 3D). It shows that each family has evolved distinct and specific Pep motifs. For example, in the Brassicaceae-specific sequence there is only one partially conserved proline, whereas proline residues seem to play an important role in the sequence of Peps from Solanaceae, and the Poaceae-specific consensus sequence is rich in glycine residues and conserved histidine residues at the terminal end of the peptides.

### Validation of novel Pep-motifs

**Fig. 4.**
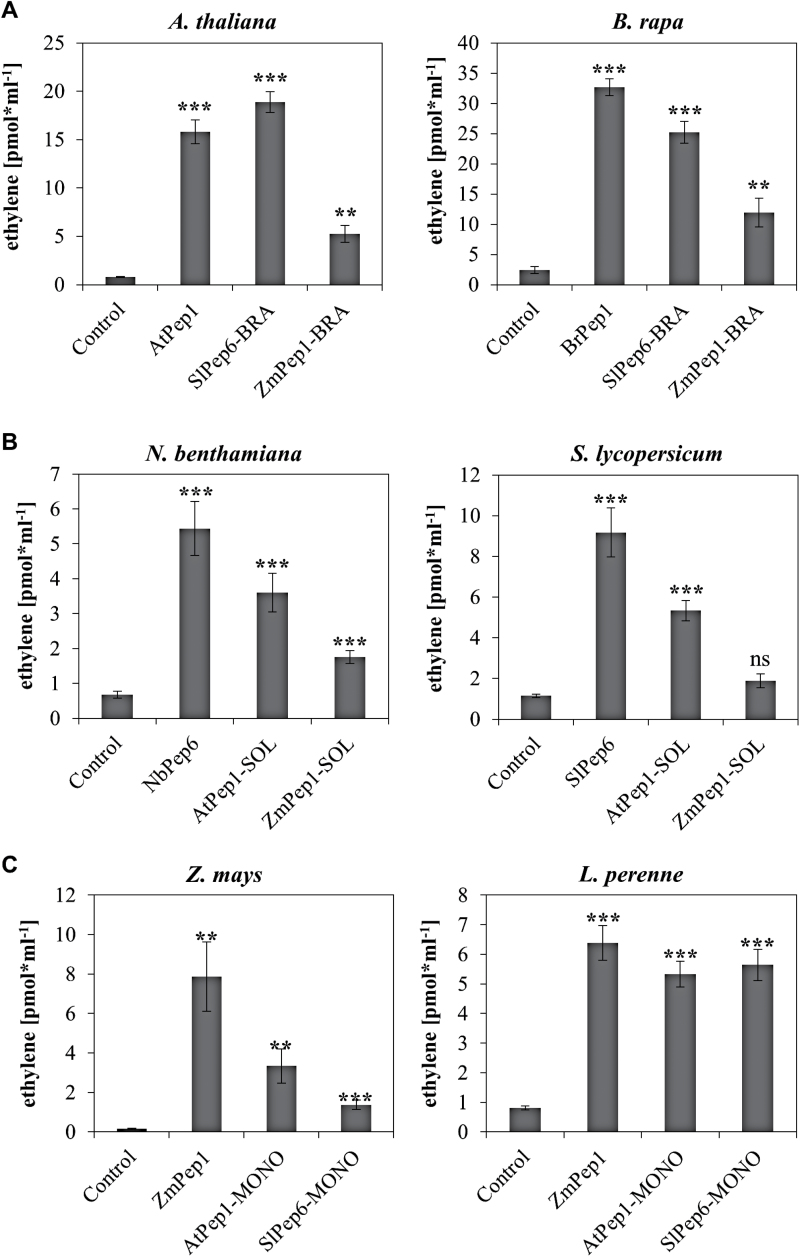
**Validation of family-specific Pep motifs with mutated Peps.** Ten leaf discs of indicated plant species (with species in A representing Brassicaceae, in B Solanaceae, and in C Poaceae) were treated for 5h with 1 µM of the indicated elicitor peptides or without any peptide (control). BRA indicates the introduction of the Brassicaceae-specific motif into the Pep sequence (A), SOL indicates the introduction of the Solanaceae-specific motif into the Pep sequence (B), and MONO marks mutated peptides containing the Poaceae (monocot)-specific motif in their sequence (C). Columns represent averages of detected ethylene values of five biological replicates. Error bars show the standard error of the mean. Asterisks indicate significant differences of the labelled column to the control based on t-test results (*P < 0.05; *P < 0.01; ***P < 0.001).

Are family-specific Pep-motifs sufficient for Pep recognition? In order to address this question, sequences of AtPep1, SlPep6, and ZmPep1 were mutated to introduce the family-specific motif of non-origin plant families, resulting in AtPep1-SOL and AtPep1-MONO [containing the motifs of the Solanaceae (SOL) and the Poaceae (MONO, monocots)], SlPep6-BRA and SlPep6-MONO [containing the Brassicaceae (BRA) and Poaceae motifs, respectively], and ZmPep1-BRA and ZmPep1-SOL (Supplementary Table S1). As demonstrated by the ethylene production of leaf tissue taken from the Brassicaceae representatives (*Arabidopsis* and *B. rapa*), these modified peptides containing the BRA-Pep motifs were recognized. Likewise, the Solanaceae and Poaceae species responded to the SOL and the MONO peptides, respectively (Fig. 4). However, despite a significant response to all peptides, the ZmPep1-BRA and ZmPep1-SOL peptides did not trigger a level of ethylene production comparable to that triggered by perception of the species-specific control peptide, indicating that additional residues outside the motifs contribute to the Pep-PEPR interaction.

### PEPRs show interfamily compatibility

**Fig. 5.**
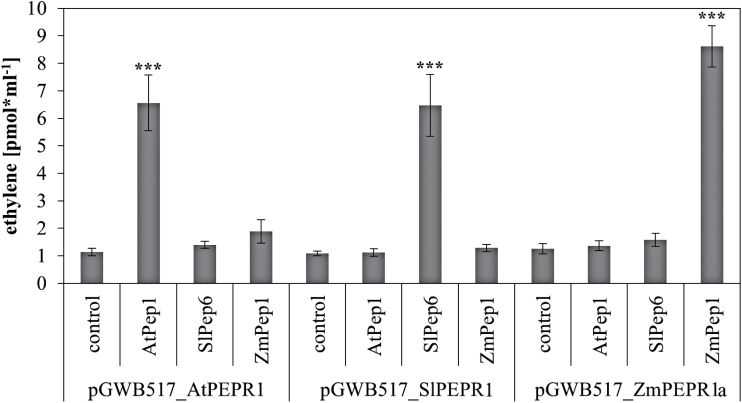
**Detection of Peps by transiently expressed PEPRs.** *N. benthamiana* plants were transiently transformed with *Agrobacteria* containing pGWB517 plasmids harbouring the coding sequences of either AtPEPR1, SlPEPR1, or ZmPEPR1a (as indicated). Leaf discs were harvested one day past transformation. Columns represent averages of detected ethylene values of six biological replicates (containing three leaf discs each) 5h after treatment with the indicated peptides or without any peptide (control). Error bars show the standard error of the mean. Asterisks indicate significant differences of the labelled column to the control based on t-test results (*P < 0.05; *P < 0.01; ***P < 0.001).

A correlation between PEPR sequence divergence and interfamily incompatibility of the system was investigated. In the first study describing AtPEPR1, the authors used the alkalinization response of transgenic tobacco cells expressing AtPEPR1 to show that AtPEPR1 recognizes AtPep1 (Yamaguchi *et al.*, 2006). Thus, at least AtPEPR1 functions also in tobacco cells and not just in Brassicaceae. Here, additional PEPRs were cloned and studied by introducing the coding sequences of AtPEPR1, the tomato PEPR SlPEPR1, and the maize PEPR ZmPEPR1a into the expression vector pGWB517 and transiently expressing them in *N. benthamiana* leaves. Again, the elevated production of ethylene was used as a read-out for the activation of PEPR signalling upon perception of Peps. Leaf tissue of *N. benthamiana* is naturally insensitive to AtPep1 and ZmPep1; however, when transformed with AtPEPR1 or ZmPEPR1, it responded with a strong production of ethylene (Fig. 5). Remarkably, in this assay no significant ethylene production was detected in SlPep6-treated leaf discs despite the previously noted sensitivity of wild-type *N. benthamiana* leaves to SlPep6 (Fig. 2 and Fig. 3B). In contrast, leaf discs transiently expressing SlPEPR1 again responded with strong ethylene production upon addition of SlPep6 (Fig. 5). This apparent discrepancy is based on the use of only three discs per replicate harvested from the transiently transformed leaves in this experiment compared to 10 discs per replicate used in the assays based on wild-type leaves. Three discs are not enough to detect the little ethylene production elicited in wild-type discs upon SlPep6 treatment but are sufficient to show the strong SlPep6-dependent production of ethylene in leaf discs transiently transformed with SlPEPR1. Thus as reported before (Flury *et al.*, 2013), the overexpression of PEPRs boosts Pep-triggered responses.

Like SlPEPR1, AtPEPR1 and ZmPEPR1a are able to activate downstream signalling pathways despite their transfer into the unrelated species *N. benthamiana*. This prompted a further analysis of the PEPR-LRR domain, which detects Peps, and the PEPR-kinase domain, which is crucial for downstream signalling. As can be seen in Supplementary Table S6 and Supplementary Table S7, the PEPR-LRR domains showed a distinctly lower sequence identity than the PEPR-kinase domains. For example, the sequence identity of AtPEPR1-LRR and SlPEPR1-LRR was 47.6% whereas the one of AtPEPR1-kinase and SlPEPR1-kinase was 55.9%. Within the large cluster of Poaceae-PEPRs the sequence identity of these PEPR-LRRs ranged between 52.4% and 89.3% whereas the sequence identity of the kinase domains ranged from 66.4% to 95.3%. These data support the idea that the kinase domain is more strictly conserved, given that it has a catalytic role and interacts with the complex defence signalling network whereas the LRR domain is not subjected to catalytic constraints but has evolved necessary plasticity to recognize specific ligands, as it has done with the Peps (PROPEPs). Based on the AtPEPR1-LRR-AtPep1 crystal structure, a number of amino acids within the LRRs LRR4 to LRR18 of the AtPEPR1-LRR domain were identified to interact with AtPep1 (Tang *et al.*, 2015). Thus, the plasticity of the Pep-PEPR interaction was further analysed by determining the conservation of the interaction site within the PEPR-LRR domain. As shown in Supplementary Figure S1, the LRR-domain sequences of all identified PEPRs were aligned and the Pep-interacting amino acids were highlighted in magenta based on the AtPEPR1-LRR. Only seven of the 25 amino acids interacting with Pep showed a considerable degree of conservation whereas the other 18 appeared not to be conserved.

Taken together, contrary to the PROPEPs, PEPRs are interfamily compatible. Their kinase domains are more strictly conserved than their LRR, including the Pep interaction site, arguably reflecting the co-evolution of the LRRs and the PROPEPs.

## Discussion

In recent years a couple of distinct endogenous signalling peptides involved in plant defence processes were reported (Albert, 2013). They were identified from different species but most of them, notably systemins, appear to be restricted to specific plant families (Ryan and Pearce, 2003; Pearce *et al.*, 2010). Two exceptions are phytosulfokines (PSKs) and rapid alkalinization factors (RALFs), which have been shown to be present in a broad range of species but only a small number of reports link them to plant defence (Pearce *et al.*, 2001; Igarashi *et al.*, 2012; Albert, 2013; Mosher *et al.*, 2013; Sauter, 2015). PSKs were classified as growth factors with additional functions in diverse developmental processes and PSK-triggered signalling was shown to negatively affect PTI (Igarashi *et al.*, 2012; Hartmann *et al.*, 2014; Sauter, 2015). Likewise, RALFs also regulate plant growth as well as other developmental processes including pollen tube elongation (Covey *et al.*, 2010; Murphy and De Smet, 2014). Their association with plant defence is based only on the induction of physiological responses that have been linked to PTI (Pearce *et al.*, 2001; Albert, 2013). Thus, even though these peptides and their dependent signalling network are currently discussed as integrators of plant growth and defence they are regarded as growth factors rather than DAMPs (Murphy and De Smet, 2014; Sauter, 2015).

In contrast, Peps have been tightly linked to plant defence and are regarded as DAMPs (Albert, 2013; Bartels and Boller, 2015). Despite their discovery in *Arabidopsis*, previous studies indicated that the Pep-PEPR system is not an invention made by the Brassicaceae but that at least PROPEPs are present in multiple species (Huffaker *et al.*, 2006; Huffaker *et al.*, 2011; Huffaker *et al.*, 2013; Trivilin *et al.*, 2014). However, the first identified and characterized ortholog of AtPeps, ZmPep1, showed extensive differences in its amino acid sequence, raising some doubts about its homology to AtPep1 (Huffaker *et al.*, 2011). But their functional similarity has been shown in a number of studies, thus the sequence diversity seems to be rather a sign for a strong divergence of the system (Huffaker *et al.*, 2006; Huffaker *et al.*, 2011; Liu *et al.*, 2013). Because a detailed analysis of the presence and activity of the Pep-PEPR system including the PEPRs has hitherto not been undertaken, the interspecies and interfamily compatibility of the system have here been analysed.

Based on the presented data it is now clear that the Pep-PEPR system is widely present within the angiosperms. Neither potential PROPEPs nor PEPRs could be identified in the gymnosperms or lower plants. This does not necessarily mean that there are no PROPEPs or PEPRs, because most sequenced plant genomes belong to species of the angiosperms and the seemingly high plasticity of the PROPEPs could mask their identification. Moreover, PEPRs likely evolved from the numerous receptors regulating plant development, which additionally exacerbates their conclusive identification in the more primordial plant species (Yamaguchi *et al.*, 2010).

Consistent with previously reported data from Huffaker *et al.* (2013), interfamily incompatibility of Peps was uncovered. Although a considerable number of Peps contained the previously identified ser^15^, gly^17^, and asp^23^ (Pearce *et al.*, 2008), it appears that contrary to the previous assumptions these residues may not be a prerequisite of Pep activity in general. The novel conserved motifs described in this study (Fig. 3D) rather point to the fact that a larger number of Pep residues are important for Pep-PEPR interaction and, with it, Pep ‘activity’. The recently resolved crystal structure of AtPEPR1-LRR in conjunction with AtPep1 supports this idea because multiple Pep residues were found to be in close contact with the PEPR1-LRR (Tang *et al.*, 2015). In addition, proline^19^ as well as glutamine^21^ and histidine^22^ seem to be crucial for the interaction of PEPR1 with its co-receptor BAK1 (Tang *et al.*, 2015). Notably, the lack of BAK1 together with its closest relative BKK1 (BAK1-LIKE1) completely impairs PEPR signalling (Roux *et al.*, 2011). In summary, no typical strictly conserved Pep motif was found and thus it is proposed that Peps and their precursors PROPEPs as well as the ligand-binding (LRR) domain of the PEPRs rapidly diverged, producing distinct Pep motifs and, as a consequence, the interfamily incompatibility. However, it is also possible that some Peps retained a rather more generic sequence and structure that is still binding loosely to LRRs from more distantly related species.

Contrary to the incompatibility of Peps, the PEPRs appear to be interfamily compatible. Transient expression of AtPEPR1 and ZmPEPR1a in *N. benthamiana* enabled AtPep1 and ZmPep1 sensitivity. In light of the higher level of conservation of the PEPR kinase domain compared to the rather variable sequence of the PEPR LRR domain, including the Pep interaction site, it seems that only the Pep detection via the LRR domain features a substantial plasticity whereas the intracellular part of the PEPR operates a strictly conserved defence signalling system. In support of this view is the involvement of BAK1 and BKK1 as co-receptors of PEPRs (Schulze *et al.*, 2010; Roux *et al.*, 2011). BAK1 in particular has been linked to numerous receptors involved in plant defence signalling and is thus regarded as a signalling hub (Chinchilla *et al.*, 2009; Roux *et al.*, 2011). In addition, an observation similar to the PEPR interfamily transfer has been made with the interfamily transfer of EF-Tu receptor (EFR), which has evolved in the Brassicaceae to detect the presence of the bacterial protein EF-Tu (Zipfel *et al.*, 2006; Lacombe *et al.*, 2010). Expression of EFR in *N. benthamiana* or *S. lycopersicum* enabled the detection of EF-Tu in both species and improved the resistance against a number of pathogenic bacteria (Lacombe *et al.*, 2010). Finally, PEPR signalling has been reported to induce jasmonic acid, salicylic acid, and ethylene-dependent genes (Ross *et al.*, 2014). Because plant immunity is constructed as a robust network where jasmonic acid, salicylic acid, and ethylene signalling significantly overlap to compensate for the loss of individual signals (Tsuda *et al.*, 2009), PEPRs seem to occupy a central and/or flexible role here. Thus there is most likely no room for plasticity of the intracellular part of the PEPRs. However, whether the plasticity of the Pep/PEPR-LRR interaction is of advantage for PEPR signalling (e.g. by evading inhibitory action of bacterial peptides) still needs to be determined.

## Conclusion

Contrary to the detection of conserved microbe-associated molecular patterns that require an equally conserved detector domain of the microbe-associated molecular pattern receptor, the sequences of Peps and PEPR-LRRs appear to evolve more dynamically, resulting in a considerable divergence of the Pep-PEPR system. The identification of the variable plant family-specific Pep motifs will probably help to uncover more PROPEPs with the advancing number of sequenced plant genomes and the improved gene annotation. Moreover, activation of the Pep-PEPR system has been shown to effectively improve resistance against a broad spectrum of pathogens, including bacteria and fungi, as well as herbivores. Having learnt that the Pep-PEPR system is common among angiosperms, two approaches could be valuable for improving cultivation of crop plants. Firstly, marker-assisted breeding should be implemented to track and conserve the Pep-PEPR system during crop plant breeding. Secondly, rationally designed synthetic Peps could be used to boost plant resistance of especially valuable crops when pathogen attack is imminent. Thus it is no surprise that integral parts of the Pep-PEPR system have already been patented.

## Supplementary data

Supplementary data are available at *JXB* online.

Supplementary Table S1: Sequences of Peps and mutated Peps used in this study.

Supplementary Table S2: Sequence information for all identified PROPEPs and PEPRs.

Supplementary Table S3: Comparison of PROPEP sequence identity.

Supplementary Table S4: Comparison of Pep sequence identity.

Supplementary Table S5: Comparison of PEPR full length sequence identity.

Supplementary Table S6: Comparison of PEPR-LRR domain sequence identity.

Supplementary Table S7: Comparison of PEPR-kinase domain sequence identity.

Supplementary Fig. S1: Analysis of the conservation of the Pep-PEPR-LRR interaction site.

Supplementary Data
