# Supplementary material for: Evolutionary divergence of the plant elicitor peptides (Peps) and their receptors: interfamily incompatibility of perception but compatibility of downstream signalling
Source: J Exp Bot. 2015 May 22;66(17):5315–25. doi: 10.1093/jxb/erv236 (PMC4526913; doi:10.1093/jxb/erv236)
Supplement: Supplementary Data [file supp_66_17_5315__index.html]

Evolutionary divergence of the plant elicitor peptides (Peps) and their receptors: interfamily incompatibility of perception but compatibility of downstream signalling — Evolutionary divergence of the plant elicitor peptides (Peps) and their receptors: interfamily incompatibility of perception but compatibility of downstream signalling — Supplementary Data 

# Evolutionary divergence of the plant elicitor peptides (Peps) and their receptors: interfamily incompatibility of perception but compatibility of downstream signalling

## Supplementary Data

Data files

**Files in this Data Supplement:**

- Supplementary Data - Supplementary Data
- Supplementary Data - Supplementary Data
- Supplementary Data - Supplementary Data
