# Supplementary material for: Evolutionary divergence of the plant elicitor peptides (Peps) and their receptors: interfamily incompatibility of perception but compatibility of downstream signalling
Source: J Exp Bot. 2015 May 22;66(17):5315–25. doi: 10.1093/jxb/erv236 (PMC4526913; doi:10.1093/jxb/erv236)
Supplement: Supplementary Data [file supp_erv236_jexbot147900_file003.pdf]

Evolutionary divergence of the plant elicitor peptides (Peps) and their receptors: interfamily incompatibility of perception but compatibility of downstream signalling

Martina Lori, Marcel van Verk, Tim Hander, Hendrik Schatowitz, Dominik Klauser, Pascale Flury, Chris Gehring, Thomas Boller, and Sebastian Bartels

Supplemental Files

Supplementary Table S3: Identity comparison of PROPEP sequences
Full-length amino-acid sequences of published and novel HMMER identified PROPEP sequences were compared for the percentage of identical residues in aligned positions (upper half), and the number of identical residues in aligned positions (lower half). Colours indicate increasing amount of identity from low (blue) via white to high (red).

Supplementary Table S4: Identity comparison of Pep sequences
Pep amino-acid sequences deduced from published and novel HMMER identified PROPEP sequences were compared for the percentage of identical residues in aligned positions (upper half), and the number of identical residues in aligned positions (lower half). Colours indicate increasing amount of identity from low (blue) via white to high (red).

Supplementary Table S5: Identity comparison of PEPR sequences
Full-length amino-acid sequences of PEPR sequences were compared for the percentage of identical residues in aligned positions (upper half), and the number of identical residues in aligned positions (lower half). Colours indicate increasing amount of identity from low (blue) via white to high (red).

Supplementary Table S6: Identity comparison of PEPR LRR domains
The LRR domains of PEPR sequences were compared for the percentage of identical residues in aligned positions (upper half), and the number of identical residues in aligned positions (lower half). Colours indicate increasing amount of identity from low (blue) via white to high (red).

Supplementary Table S7: Identity comparison of PEPR kinase domains
The kinase domains of PEPR sequences were compared for the percentage of identical residues in aligned positions (upper half), and the number of identical residues in aligned positions (lower half). Colours indicate increasing amount of identity from low (blue) via white to high (red).

Supplementary Figure S1: Plasticity of the Pep-PEPR-LRR interaction site
Sequence alignment of the LRR region of the PEPR sequences from Figure 1B. Amino acid residues are coloured based on the conservation and interacting residues of AtPEPR1-LRR (underlined in red) as described by Tang et al., 2015. The LRR sequence of SlPEPR1 and ZmPEPR1a, used in this study, are underlined in grey. Yellow residues: conserved amino acids of AtPEPR1LRR. Magenta residues: AtPep1 interacting amino acids of AtPEPR1LRR. Below the sequence alignment the overall conservation is indicated by pink bars and a sequence logo.

		1	2	3	4	5	6	7	8	9	10	11	12	13	14	15	16	17	18	19	20	21	22	23	24	25	26	27	28	29	30	31	32	33	34	35	36	37	38	39	40	41	42	43	44	45	46	47	48	49	50	51	52	53	54	55	56	57	58	59	60	61	62	63	64	65	66	67	68	69	70	71	72	73	74	75
AlPROPEP2b	1		98.21	59.83	57.26	58.33	47.62	38.21	35.97	37.80	34.38	36.64	25.21	25.21	24.37	22.69	19.53	21.67	18.85	20.49	13.60	13.60	17.60	17.60	16.80	5.15	5.56	4.69	7.26	6.82	6.92	8.57	8.27	7.45	14.69	14.69	15.28	15.17	15.60	15.60	11.81	13.19	6.06	8.24	5.62	4.92	5.26	5.14	4.33	3.46	4.29	5.18	4.57	6.25	6.25	6.31	4.76	4.76	4.88	3.76	3.90	2.39	6.32	6.74	5.78	5.38	7.07	4.09	8.67	10.14	10.77	1.93	2.68	4.14	7.95	9.42
AlPROPEP2c	2	110		59.83	58.12	59.17	46.83	37.40	35.25	37.80	33.59	35.88	25.21	25.21	24.37	22.69	19.53	21.67	18.85	20.49	13.60	13.60	17.60	17.60	16.00	5.15	5.56	4.69	7.26	6.82	6.92	8.57	8.27	7.45	14.69	14.69	15.28	15.17	15.60	15.60	11.81	13.19	6.06	8.24	5.62	4.92	5.26	5.14	4.33	3.46	4.29	5.18	4.57	6.73	6.73	6.76	4.76	4.76	4.88	3.76	4.33	2.39	6.32	6.74	5.78	5.38	7.07	4.09	8.67	10.14	10.77	1.93	2.68	4.14	7.95	9.42
AlPROPEP2a	3	70	70		72.73	54.78	43.33	39.32	35.34	40.16	36.07	37.30	26.89	26.05	26.05	23.48	22.31	21.67	23.48	24.35	18.33	18.49	19.51	20.33	17.89	6.28	6.70	5.76	8.57	8.14	10.32	11.76	10.85	9.49	17.39	17.39	17.27	17.86	18.25	19.15	13.38	13.38	8.59	10.12	6.36	6.67	5.80	5.66	5.24	4.37	4.76	6.81	6.15	6.80	6.80	6.82	5.77	5.77	5.91	5.21	4.37	3.09	7.56	7.95	5.85	5.43	6.59	5.33	8.19	8.76	10.85	1.54	3.15	5.63	10.14	10.49
AtPROPEP2	4	67	68	80		53.04	45.83	40.17	36.09	35.25	31.97	38.89	25.21	24.37	25.21	23.48	22.31	21.67	23.48	25.22	16.67	16.81	18.70	19.51	18.70	5.76	6.18	5.26	7.47	7.60	8.44	10.37	8.59	7.64	17.52	17.52	18.84	16.55	17.65	19.29	13.48	12.77	6.79	8.38	5.23	5.59	5.34	5.21	4.39	3.51	4.35	5.26	4.64	5.85	5.85	6.39	4.83	4.83	4.95	4.29	4.39	2.76	7.60	7.43	5.29	6.01	6.08	4.14	8.19	8.82	11.72	2.32	4.07	5.63	8.78	10.56
CrPROPEP2	5	70	71	63	61		42.28	34.71	35.07	34.13	32.54	35.43	23.53	23.53	22.69	20.69	18.03	20.83	18.97	18.97	14.17	14.17	16.39	16.39	18.03	5.76	6.59	5.67	9.55	9.71	8.86	10.79	11.36	10.00	16.20	16.20	17.48	16.67	14.89	15.97	10.96	13.01	4.79	7.56	6.21	5.43	4.74	4.63	3.86	3.43	4.26	5.13	4.52	4.76	4.76	5.80	5.19	5.19	5.31	4.65	5.15	2.37	6.82	6.67	5.14	4.26	7.53	4.12	10.47	10.00	12.88	2.67	3.54	7.09	8.78	8.22
EsPROPEP1	6	60	59	52	55	52		50.00	49.58	54.13	54.13	45.08	27.43	27.43	25.66	25.69	24.14	23.48	20.72	20.72	19.13	19.30	20.51	21.37	21.19	8.43	8.72	7.61	9.94	10.24	8.78	12.98	12.90	9.93	19.55	18.80	20.15	17.65	14.29	18.25	13.14	13.87	8.86	10.43	7.10	7.47	7.46	7.28	5.83	5.38	5.33	6.45	6.32	5.50	5.50	7.01	5.45	5.45	5.58	4.88	4.91	2.80	5.99	7.60	7.23	5.59	6.78	2.45	7.83	10.29	11.72	1.96	3.69	6.20	13.43	8.61
AtPROPEP1	7	47	46	46	47	42	54		63.79	42.20	44.04	47.83	26.36	26.36	24.55	27.88	23.21	27.27	24.30	24.07	16.22	16.36	19.47	19.47	18.42	9.71	9.76	8.52	13.17	13.66	11.97	14.17	15.00	11.64	18.25	18.11	19.05	19.69	16.94	18.75	14.96	15.62	10.88	9.21	6.29	6.06	7.45	7.25	5.71	5.21	5.66	5.65	5.52	7.45	7.45	7.43	5.70	5.70	5.85	4.64	5.63	3.66	8.92	8.12	7.74	7.60	7.10	3.27	8.33	10.48	13.68	1.63	3.38	7.94	17.05	10.79
CrPROPEP1	8	50	49	47	48	47	59	74		47.06	49.58	52.46	23.58	23.58	21.95	20.17	20.63	24.39	19.17	21.67	19.01	19.17	20.97	20.97	20.97	10.06	9.50	7.85	10.61	10.92	9.49	11.19	11.03	11.04	17.14	17.73	17.14	19.15	13.77	15.49	11.03	12.41	9.04	8.19	5.65	5.46	6.64	6.48	5.15	4.29	4.26	6.19	5.56	5.24	5.71	5.36	5.61	5.61	5.74	4.61	5.60	2.72	6.29	7.82	8.05	6.32	4.81	1.83	5.95	9.29	11.85	1.89	4.00	9.77	14.60	8.18
BnPROPEP1	9	48	48	49	43	43	59	46	56		87.04	44.26	25.86	25.86	25.00	25.00	22.50	23.93	22.81	24.56	18.49	18.64	19.67	19.67	18.03	6.67	7.51	6.49	11.76	12.12	9.27	10.45	10.24	8.44	16.79	16.06	16.67	15.83	13.33	15.71	14.07	12.59	8.12	9.70	6.98	6.78	7.96	7.77	5.83	5.83	5.78	7.61	7.45	6.00	6.50	7.94	6.86	6.86	7.04	5.80	5.86	4.23	7.88	8.88	8.54	7.78	5.08	3.03	7.93	10.53	12.80	1.97	3.72	6.77	11.68	6.04
BrPROPEP1b	10	44	43	44	39	41	59	48	59	94		45.90	26.50	26.50	25.64	23.89	22.50	23.73	20.18	22.81	20.17	20.34	21.31	21.31	21.31	8.24	9.14	7.49	13.53	13.94	10.60	11.19	11.02	8.44	18.98	18.25	19.57	18.71	13.97	16.43	13.14	12.41	7.45	9.04	6.40	6.18	7.88	7.69	5.78	5.78	5.73	6.99	6.84	4.95	5.45	6.94	7.28	7.28	7.46	6.22	5.80	3.85	7.19	8.19	8.43	7.69	5.03	2.99	8.43	8.89	13.39	2.34	4.15	8.27	13.14	4.64
BrPROPEP1a	11	48	47	47	49	45	55	55	64	54	56		26.67	25.00	25.83	25.86	23.58	25.62	21.37	20.51	19.33	19.33	20.49	18.85	19.67	6.08	6.29	5.88	10.29	10.00	9.80	12.32	12.21	8.86	19.12	19.85	21.17	18.84	15.56	17.99	13.29	13.29	6.13	7.74	5.75	6.11	8.61	8.41	4.76	5.19	5.15	7.29	7.14	5.77	5.77	7.21	6.16	6.16	6.31	5.61	5.22	3.08	6.94	7.34	5.81	5.35	7.07	3.68	8.33	12.69	13.18	1.92	3.59	9.70	10.71	7.19
AaPROPEP4	12	30	30	32	30	28	31	29	29	30	31	32		95.18	90.36	70.93	61.05	65.17	32.63	36.84	26.53	26.80	34.69	33.67	31.63	6.90	6.63	6.82	8.93	8.38	6.80	10.61	12.10	8.50	17.89	17.89	18.55	15.75	13.82	17.07	11.29	10.85	8.84	8.55	5.62	6.71	4.76	4.64	3.29	3.76	3.76	3.45	3.37	4.26	4.26	4.48	4.66	4.66	4.79	3.57	4.19	2.90	6.45	6.33	5.88	4.22	7.32	5.48	5.81	11.20	8.77	1.65	2.40	8.40	7.75	8.89
AlPROPEP4	13	30	30	31	29	28	31	29	29	30	31	30	79		87.80	66.28	57.89	64.04	31.58	34.74	25.51	25.77	33.67	32.65	32.65	6.32	6.02	6.25	8.98	8.43	6.85	9.09	11.29	8.50	17.89	17.89	18.55	15.75	13.82	17.07	11.29	10.85	8.90	8.55	5.62	6.71	4.76	4.64	2.82	3.29	3.76	3.47	3.39	4.26	4.26	4.48	4.15	4.15	4.26	3.57	4.19	2.90	6.49	6.37	5.92	4.24	7.36	5.52	5.81	10.48	8.85	1.65	2.88	8.47	7.81	8.96
AtPROPEP4	14	29	29	31	30	27	29	27	27	29	30	31	75	72		65.12	54.74	59.55	30.53	33.68	24.49	24.74	30.61	30.61	29.59	6.36	6.02	5.68	9.64	9.09	7.59	10.61	12.10	8.50	16.39	16.39	17.07	14.17	13.93	17.21	11.29	10.85	8.97	8.55	5.62	6.71	4.76	4.64	2.82	3.29	3.76	3.49	3.41	4.26	4.26	4.48	4.15	4.15	4.26	3.59	4.19	2.54	6.54	6.41	5.96	4.27	7.41	5.56	5.16	12.20	8.93	1.65	2.40	7.69	7.87	8.27
CrPROPEP4	15	27	27	27	27	24	28	29	24	28	27	30	61	57	56		53.19	56.52	30.11	33.33	24.47	24.73	29.90	28.87	27.84	5.26	5.52	5.78	8.54	8.02	6.25	8.59	10.00	6.67	15.70	15.70	17.21	14.40	13.22	16.00	11.76	10.48	7.59	8.05	5.70	6.79	4.37	4.26	3.38	3.37	2.90	2.94	2.87	3.83	4.37	4.59	4.79	4.79	4.92	3.16	3.35	3.33	6.67	5.88	5.41	6.21	6.29	4.26	5.30	9.09	8.26	1.67	3.45	6.72	7.75	9.02
EsPROPEP4	16	25	25	27	27	22	28	26	26	27	27	29	58	55	52	50		56.70	30.53	35.42	24.24	24.49	29.41	28.43	30.39	6.94	6.06	6.21	7.74	7.23	5.48	10.16	10.92	7.48	19.35	20.16	18.25	17.05	14.29	16.92	13.01	11.72	9.15	7.28	6.25	6.67	4.28	4.17	3.35	3.35	3.32	3.35	3.28	3.19	3.19	3.98	4.17	4.17	4.28	3.61	4.21	3.64	7.59	6.83	6.41	5.92	7.32	4.93	7.33	11.11	8.62	2.50	2.91	8.94	8.21	9.79
BrPROPEP4	17	26	26	26	26	25	27	30	30	28	28	31	58	57	53	52	55		32.63	38.95	24.49	24.74	31.63	30.61	27.27	5.75	5.42	5.62	6.98	6.43	6.62	6.82	8.87	5.88	15.87	15.87	17.46	16.54	13.82	17.07	12.60	12.31	6.85	7.28	5.66	6.10	4.19	4.08	2.35	2.35	3.26	3.35	3.28	4.15	4.15	4.37	3.11	3.11	3.19	3.06	2.79	2.90	6.29	5.56	5.10	5.29	6.55	6.90	8.44	9.38	8.55	2.05	2.39	9.84	6.02	9.29
AtPROPEP8	18	23	23	27	27	22	23	26	23	26	23	25	31	30	29	28	29	31		67.07	19.79	20.00	21.43	21.21	21.43	8.05	9.20	9.14	12.80	11.04	9.15	14.63	12.07	13.19	19.33	19.33	17.50	16.39	16.81	17.89	13.33	13.49	6.99	6.71	5.84	7.45	5.43	5.29	4.85	4.37	4.33	4.68	4.57	4.30	4.30	5.56	5.32	5.32	5.46	4.21	3.35	4.09	6.62	6.49	7.38	6.17	6.88	5.63	10.14	9.68	10.71	1.68	3.47	7.50	9.92	8.89
EsPROPEP8	19	25	25	28	29	22	23	26	26	28	26	24	35	33	32	31	34	37	55		22.92	23.16	26.53	26.26	22.45	10.92	11.04	10.86	12.20	9.82	7.69	13.82	11.21	9.66	21.01	21.01	19.17	20.49	16.81	17.89	10.74	11.11	7.64	8.05	4.55	6.83	4.30	4.19	3.85	3.37	3.33	3.49	3.41	4.26	4.26	5.00	3.72	3.72	3.83	3.14	2.38	3.33	5.92	6.45	7.33	6.13	6.21	8.39	10.07	8.00	9.73	2.09	3.94	5.74	7.52	7.30
AaPROPEP7a	20	17	17	22	20	17	22	18	23	22	24	23	26	25	24	23	24	24	19	22		98.84	73.63	74.73	58.06	5.23	3.64	3.39	6.51	5.36	3.97	10.00	9.76	5.30	16.53	16.39	17.36	17.46	11.48	14.29	8.06	8.73	6.80	7.19	3.77	6.06	2.69	2.62	2.88	2.88	3.33	2.87	2.81	2.66	2.66	4.48	3.19	3.19	3.28	4.19	3.35	2.94	3.25	3.82	3.29	4.24	4.32	4.93	5.23	6.30	6.96	2.50	4.90	7.32	5.84	7.97
AtPROPEP7	21	17	17	22	20	17	22	18	23	22	24	23	26	25	24	23	24	24	19	22	85		73.63	74.73	58.06	5.23	3.64	3.39	6.51	5.36	3.97	10.00	9.76	5.30	16.67	16.53	17.50	17.60	11.57	14.40	8.13	8.80	6.85	7.24	3.80	6.10	2.70	2.63	2.90	2.90	3.35	2.89	2.82	2.67	2.67	4.50	3.21	3.21	3.30	4.21	3.37	2.95	3.27	3.85	3.31	4.27	4.35	4.96	5.26	6.30	6.96	2.50	4.93	7.32	5.88	7.97
AaPROPEP7b	22	22	22	24	23	20	24	22	26	24	26	25	34	33	30	29	30	31	21	26	67	67		94.44	65.93	5.75	4.17	3.89	6.94	5.81	3.95	9.02	8.73	5.84	16.13	16.00	16.94	17.83	10.40	15.08	7.03	7.69	5.96	5.77	2.47	4.76	2.12	2.06	1.90	1.90	2.82	2.23	2.19	2.62	2.62	3.92	2.60	2.60	2.67	3.59	3.29	2.55	3.14	3.09	2.55	3.53	3.59	4.14	5.73	6.06	7.50	2.90	4.33	8.26	5.93	5.71
AlPROPEP7	23	22	22	25	24	20	25	22	26	24	26	23	33	32	30	28	29	30	21	26	68	68	85		64.52	5.75	4.17	3.89	6.94	5.81	3.90	9.77	8.73	6.49	16.13	16.00	16.94	17.83	10.40	15.08	7.03	7.69	5.96	5.77	2.47	4.76	2.11	2.05	2.36	1.89	2.80	2.23	2.19	2.60	2.60	3.90	2.60	2.60	2.67	3.59	3.27	2.54	3.14	3.09	2.55	3.53	3.59	4.14	5.73	6.82	7.50	2.90	4.33	7.32	5.84	6.43
CrPROPEP7	24	21	20	22	23	22	25	21	26	22	26	24	31	32	29	27	31	27	21	22	54	54	60	60		5.71	5.33	4.97	5.81	5.26	5.26	8.96	8.66	7.74	15.20	15.87	16.80	16.15	9.52	13.39	6.25	6.92	5.96	5.10	3.68	5.33	2.62	2.55	1.88	2.35	2.33	2.81	2.75	3.12	3.12	3.90	3.09	3.09	3.17	4.06	3.74	2.55	4.43	4.35	3.85	3.55	4.22	4.08	5.13	8.40	7.56	2.48	3.85	6.50	5.93	6.47
NbPROPEP6	25	10	10	12	11	11	15	17	18	12	15	11	12	11	11	9	12	10	14	19	9	9	10	10	10		73.33	64.37	34.38	31.77	30.06	19.54	18.50	23.73	13.45	13.95	14.71	12.14	13.53	14.29	7.87	9.50	11.76	15.03	11.73	10.81	4.17	4.17	4.37	4.80	3.67	3.33	3.26	4.46	4.89	5.22	7.08	7.08	6.60	7.01	3.75	2.72	9.24	9.09	10.27	10.70	9.19	5.59	7.98	8.57	7.47	2.79	2.77	4.55	12.15	2.48
NsPROPEP6	26	10	10	12	11	12	15	16	17	13	16	11	11	10	10	9	10	9	15	18	6	6	7	7	9	121		81.88	36.99	37.65	35.95	20.51	20.26	25.79	14.65	15.19	15.92	13.21	14.94	15.72	8.81	10.62	10.26	15.29	13.66	12.88	5.05	5.05	5.21	5.21	4.02	3.63	3.54	4.85	5.31	5.34	7.61	7.61	7.11	6.50	4.13	3.33	8.93	10.18	10.78	12.57	10.30	7.06	9.09	8.18	6.83	2.59	2.97	3.93	10.75	2.69
NtPROPEP6	27	9	9	11	10	11	14	15	15	12	14	11	12	11	10	10	11	10	16	19	6	6	7	7	9	112	122		42.35	40.35	33.54	19.39	18.18	24.40	15.24	15.76	16.56	14.02	14.81	14.97	8.54	10.30	11.88	16.56	13.33	11.66	5.45	5.45	5.69	6.64	4.46	3.57	3.54	5.21	5.66	5.77	7.54	7.54	7.04	6.44	4.95	4.03	8.93	10.18	11.98	12.20	11.52	7.74	9.55	9.15	7.78	2.18	2.90	3.74	10.26	2.56
SlPROPEP6	28	13	13	15	13	17	17	22	19	20	23	18	15	15	16	14	13	12	21	20	11	11	12	12	10	66	64	72		82.19	50.00	25.00	22.44	25.15	14.38	15.00	14.38	13.66	14.38	15.85	12.50	12.99	9.94	15.06	7.69	9.36	3.92	3.86	5.09	4.17	3.49	3.12	3.11	3.38	4.33	4.72	5.34	5.34	5.39	4.78	6.64	5.36	9.82	9.04	10.43	11.90	12.50	6.36	7.95	9.03	7.74	1.46	3.77	3.95	10.99	3.24
StPROPEP1	29	12	12	14	13	17	17	22	19	20	23	17	14	14	15	13	12	11	18	16	9	9	10	10	9	61	64	69	120		57.75	22.29	21.71	23.64	13.66	13.66	12.88	12.12	12.42	13.94	12.26	12.66	9.15	13.10	9.25	9.25	3.88	3.83	4.59	3.67	3.48	3.08	3.08	3.43	4.39	4.31	5.74	5.74	5.80	4.76	6.70	5.02	9.04	8.88	10.24	12.79	11.63	5.68	6.70	7.59	6.37	1.81	3.72	4.12	12.00	3.30
SmPROPEP1	30	11	11	16	13	14	13	17	15	14	16	15	10	10	11	9	8	10	13	11	6	6	6	6	8	52	55	54	74	82		19.55	20.93	25.35	9.72	10.42	9.66	8.16	10.49	11.56	11.18	10.46	6.80	13.25	7.01	7.36	4.04	3.98	3.27	3.27	3.56	3.74	3.70	4.12	4.10	4.41	4.50	4.50	4.55	4.04	5.00	3.64	8.54	8.38	7.32	9.36	10.78	6.55	8.38	9.03	9.79	1.11	2.10	5.04	14.48	3.59
AtPROPEP6	31	12	12	16	14	15	17	18	16	14	15	17	14	12	14	11	13	9	18	17	13	13	12	13	12	34	32	32	39	35	26		72.12	32.82	16.67	16.67	16.67	16.54	15.79	18.25	16.42	20.30	8.67	13.25	8.44	11.11	4.74	4.64	3.32	3.32	4.63	4.42	3.76	2.53	2.53	3.38	7.37	7.37	7.57	7.22	4.59	3.97	6.92	7.41	7.64	7.65	9.09	3.80	8.64	8.96	9.30	1.57	2.27	5.04	10.96	1.91
BrPROPEP6	32	11	11	14	11	15	16	18	15	13	14	16	15	14	15	12	13	11	14	13	12	12	11	11	11	32	31	30	35	33	27	75		30.77	17.32	17.32	17.05	14.62	14.96	17.56	14.62	17.69	6.85	13.70	7.14	9.38	5.29	5.18	3.33	3.81	3.76	3.89	3.80	3.12	3.12	3.96	6.35	6.35	6.52	5.70	4.67	3.66	6.92	7.41	7.64	7.65	7.88	4.46	9.94	8.46	8.80	1.20	1.85	4.58	12.32	2.01
VvPROPEP6	33	12	12	15	12	16	15	17	18	13	13	14	13	13	13	10	11	9	19	14	8	8	9	10	12	42	41	41	42	39	36	43	40		13.73	14.38	14.29	10.97	13.55	13.92	11.04	13.38	8.00	14.01	12.03	11.52	3.61	3.61	3.81	3.33	3.64	3.74	3.65	4.00	4.00	3.33	5.76	5.76	5.76	6.67	4.55	3.56	13.84	13.58	11.95	10.53	10.30	2.86	8.29	7.28	7.53	1.11	2.10	2.58	11.11	5.20
AaPROPEP3	34	21	21	24	24	23	26	23	24	23	26	26	22	22	20	19	24	20	23	25	20	20	20	20	19	23	23	25	23	22	14	22	22	21		96.88	85.57	66.02	55.45	59.05	12.40	15.08	15.38	16.67	12.77	12.24	5.88	5.73	4.90	5.39	5.61	5.11	5.08	4.86	5.35	6.00	6.88	6.88	7.07	5.73	5.56	4.81	10.19	9.94	9.62	7.50	8.48	5.00	7.10	10.94	11.02	3.77	4.04	8.15	14.08	8.78
AtPROPEP3	35	21	21	24	24	23	25	23	25	22	25	27	22	22	20	19	25	20	23	25	20	20	20	20	20	24	24	26	24	22	15	22	22	22	93		84.69	64.42	54.90	58.49	12.30	14.96	15.27	16.55	11.97	11.49	5.85	5.70	4.88	5.37	5.58	5.08	5.06	4.84	5.32	5.97	6.84	6.84	7.03	5.70	5.53	4.80	10.13	9.88	9.55	7.45	8.43	4.96	7.05	10.85	10.94	3.75	4.02	8.15	14.08	8.72
AlPROPEP3	36	22	22	24	26	25	27	24	24	23	27	29	23	23	21	21	23	22	21	23	21	21	21	21	21	25	25	27	23	21	14	22	22	22	83	83		70.59	56.44	60.95	13.22	16.00	16.15	16.67	12.86	12.24	5.88	5.73	4.41	4.90	5.61	5.11	5.08	4.84	5.32	5.97	6.91	6.91	7.10	5.76	5.56	4.81	10.90	10.00	9.03	7.55	7.93	5.00	6.45	10.94	11.02	3.77	4.04	8.03	13.19	8.05
CrPROPEP3	37	22	22	25	23	24	24	25	27	22	26	26	20	20	18	18	22	21	20	25	22	22	23	23	21	21	21	23	22	20	12	22	19	17	68	67	72		50.98	56.60	14.52	16.41	17.56	18.12	10.71	11.49	5.76	5.61	4.33	5.29	5.48	4.97	5.49	5.24	6.22	5.83	5.70	5.70	5.85	4.57	4.52	4.01	9.88	8.43	8.07	7.32	6.51	5.67	7.10	10.85	10.85	3.29	3.96	6.47	12.33	7.74
BrPROPEP3	38	22	22	25	24	21	19	21	19	18	19	21	17	17	17	16	18	17	20	20	14	14	13	13	12	23	23	24	23	20	15	21	19	21	56	56	57	52		73.27	14.88	13.60	13.18	18.38	12.14	10.81	6.88	6.70	4.85	4.85	5.53	4.49	5.03	5.38	5.85	6.50	7.37	7.37	7.57	6.22	5.56	3.73	9.49	9.26	8.28	7.50	9.09	5.11	9.21	11.20	12.80	4.17	5.03	7.41	11.27	6.08
EsPROPEP3	39	22	22	27	27	23	25	24	22	22	23	25	21	21	21	20	22	21	22	22	18	18	19	19	17	25	25	25	26	23	17	25	23	22	62	62	64	60	74		14.96	16.03	14.29	19.29	12.50	11.84	6.19	6.03	4.74	4.74	4.95	4.92	4.89	4.71	5.18	5.83	6.63	6.63	6.81	6.53	5.43	4.38	9.76	9.52	8.59	7.23	8.19	5.59	10.13	11.45	12.21	4.51	4.39	8.09	12.68	6.67
CsPROPEP5	40	17	17	19	19	16	18	19	16	19	18	19	14	14	14	14	16	16	16	13	10	10	9	9	8	14	14	14	19	19	17	22	19	17	15	15	16	18	18	19		40.57	10.37	13.48	9.87	10.26	11.11	10.80	8.51	8.95	8.59	10.37	10.91	8.43	8.38	7.57	9.50	9.50	9.77	8.79	6.44	6.27	13.48	12.41	10.71	9.59	12.16	7.30	14.71	12.93	16.07	5.45	6.45	6.04	7.69	6.43
RcPROPEP5	41	19	19	19	18	19	19	20	18	17	17	19	14	14	14	13	15	16	17	14	11	11	10	10	9	17	17	17	20	20	16	27	23	21	19	19	20	21	17	21	43		14.39	16.31	11.84	12.10	7.95	7.73	5.70	6.15	6.00	6.40	6.36	7.65	7.61	7.29	6.74	6.74	6.94	6.08	7.00	4.28	13.89	12.84	12.59	9.21	11.11	5.52	12.86	10.57	14.29	5.26	6.81	7.05	8.18	6.85
PtPROPEP	42	10	10	14	11	8	14	16	15	13	12	10	13	13	13	11	13	10	10	11	10	10	9	9	9	20	16	19	16	15	10	13	10	12	20	20	21	23	17	19	14	20		30.00	21.80	24.64	6.67	7.07	6.81	6.81	5.80	5.08	5.06	5.08	5.06	5.13	6.56	6.56	6.67	5.43	6.64	4.12	9.40	9.87	9.40	7.95	9.21	7.63	7.64	8.33	9.92	2.55	4.33	5.77	8.28	6.96
RcPROPEP	43	14	14	17	14	13	17	14	14	16	15	13	13	13	13	12	11	11	10	12	11	11	9	9	8	26	24	27	25	22	20	20	20	22	23	23	23	25	25	27	19	23	39		24.65	27.89	6.45	6.32	6.47	5.45	5.63	4.79	5.32	8.47	7.89	7.54	7.57	7.57	7.69	5.82	7.58	6.06	10.76	11.18	11.39	10.00	10.56	7.45	10.78	7.95	12.00	2.33	2.73	5.36	8.88	5.68
GaPROPEP	44	10	10	11	9	11	12	10	10	12	11	10	9	9	9	9	10	9	9	7	6	6	4	4	6	21	22	22	13	16	11	13	11	19	18	17	18	15	17	18	15	18	29	35		59.26	7.18	7.18	7.43	7.43	6.31	5.85	5.82	6.77	6.70	7.28	11.58	11.58	11.58	8.76	6.94	5.56	10.13	10.49	10.06	9.94	12.20	6.92	8.05	4.38	8.86	2.68	2.63	4.65	9.25	3.83
TcPROPEP	45	9	9	12	10	10	13	10	10	12	11	11	11	11	11	11	11	10	12	11	10	10	8	8	9	20	21	19	16	16	12	18	15	19	18	17	18	17	16	18	16	19	34	41	80		5.13	5.13	7.54	5.53	5.50	6.42	6.42	5.15	5.10	5.47	8.33	8.33	8.33	6.15	5.14	5.26	8.81	9.43	9.49	8.23	9.94	8.33	8.09	4.85	8.59	1.90	3.03	3.93	8.94	4.30
SiPROPEP1a	46	11	11	12	11	10	15	14	14	16	16	18	9	9	9	8	8	8	10	8	5	5	4	4	5	9	10	11	8	8	8	9	10	7	11	11	11	11	13	12	19	14	12	12	14	10		96.77	46.37	51.11	47.25	40.91	41.34	32.04	31.84	35.16	28.08	27.59	28.14	27.09	21.50	20.72	11.43	12.57	12.57	10.00	11.17	2.11	7.49	6.82	6.47	7.76	11.52	8.82	5.88	5.26
SiPROPEP1b	47	11	11	12	11	10	15	14	14	16	16	18	9	9	9	8	8	8	10	8	5	5	4	4	5	9	10	11	8	8	8	9	10	7	11	11	11	11	13	12	19	14	13	12	14	10	150		48.04	52.22	45.99	43.75	44.13	31.52	31.32	34.59	28.08	27.59	27.59	27.09	22.90	20.72	11.43	12.57	12.57	10.00	11.17	2.05	7.29	6.63	6.29	7.60	11.26	8.61	5.74	5.13
SbPROPEP2	48	10	10	12	10	9	13	12	12	13	13	11	7	6	6	7	7	5	10	8	6	6	4	5	4	10	11	12	11	10	7	7	7	8	10	10	9	9	10	10	16	11	13	13	15	15	83	86		70.35	37.50	30.41	31.28	25.65	25.65	27.60	23.47	23.47	23.58	21.13	20.72	19.60	7.45	8.51	9.04	7.98	8.47	2.51	7.43	4.55	4.64	8.00	8.85	6.14	3.98	3.74
ZmPROPEP2	49	8	8	10	8	8	12	11	10	13	13	12	8	7	7	7	7	5	9	7	6	6	4	4	5	11	11	14	9	8	7	7	8	7	11	11	10	11	10	10	17	12	13	11	15	11	92	94	121		37.63	32.99	34.36	31.25	30.21	32.64	22.79	22.79	22.90	21.86	22.32	19.05	8.42	9.47	10.53	8.42	7.85	2.49	8.33	4.00	5.10	8.73	9.65	6.58	3.96	4.17
SbPROPEP3	50	10	10	11	10	10	12	12	10	13	13	12	8	8	8	6	7	7	9	7	7	7	6	6	5	9	9	10	8	8	8	10	8	8	12	12	12	12	12	11	17	12	12	12	14	12	86	86	72	73		30.15	31.34	34.39	33.51	34.92	22.02	21.56	22.07	21.00	18.39	19.52	9.28	10.88	9.33	7.43	9.45	3.79	8.06	5.42	6.22	6.10	6.42	5.70	5.26	4.69
ZmPROPEP1b	51	10	10	13	10	10	12	10	12	14	13	14	6	6	6	5	6	6	8	6	5	5	4	4	5	7	7	7	6	6	7	8	7	7	9	9	9	9	8	9	17	11	9	9	11	12	72	77	59	64	60		90.28	26.46	25.93	29.02	22.77	22.77	22.89	21.46	19.37	17.37	12.35	12.88	12.88	8.43	10.49	2.19	7.18	6.41	6.58	6.85	9.95	8.90	5.79	6.43
ZmPROPEP1	52	9	9	12	9	9	12	10	11	14	13	14	6	6	6	5	6	6	8	6	5	5	4	4	5	7	7	7	6	6	7	7	7	7	9	9	9	10	9	9	18	11	9	10	11	12	74	79	61	67	63	130		27.81	26.74	30.73	24.51	24.51	24.63	23.30	20.81	17.83	12.05	12.57	12.57	8.93	10.71	2.70	8.20	6.79	6.33	7.29	10.43	8.72	5.67	6.25
ZmPROPEP1a	53	13	14	14	12	10	11	14	11	12	10	12	8	8	8	7	6	8	8	8	5	5	5	5	6	10	10	11	7	7	8	5	6	8	9	9	9	10	10	9	15	14	9	16	13	10	58	58	49	60	65	50	52		91.33	51.15	17.54	17.06	17.31	16.11	16.51	15.14	8.29	7.53	7.61	3.68	5.76	3.59	5.97	5.98	5.65	7.56	7.44	6.28	4.85	5.21
ZmPROPEP3	54	13	14	14	12	10	11	14	12	13	11	12	8	8	8	8	6	8	8	8	5	5	5	5	6	11	11	12	9	9	8	5	6	8	10	10	10	12	11	10	15	14	9	15	13	10	57	57	49	58	63	49	50	137		51.43	16.11	15.64	15.87	15.17	16.51	15.54	8.20	7.49	7.57	4.17	5.70	3.06	5.45	5.41	5.62	7.14	7.44	6.25	4.83	5.18
SbPROPEP1	55	14	15	15	14	13	15	15	12	17	15	16	9	9	9	9	8	9	11	10	9	9	8	8	8	12	11	12	10	9	9	7	8	7	12	12	12	12	13	12	14	14	10	15	15	11	64	64	53	63	66	56	59	89	90		20.47	20.00	20.28	19.07	18.98	17.67	10.33	10.33	9.24	8.60	8.56	3.50	6.53	6.19	5.88	9.20	9.42	6.88	5.50	4.93
OsiPROPEP1	56	10	10	12	10	11	11	11	12	14	15	13	9	8	8	9	8	6	10	7	6	6	5	5	6	15	15	15	11	12	9	14	12	11	13	13	13	11	14	13	17	12	12	14	22	16	57	57	50	49	48	46	50	37	34	44		99.37	96.23	73.78	30.00	21.12	11.11	11.49	11.05	11.73	10.00	3.57	8.63	5.35	6.18	6.32	7.69	7.44	5.12	4.52
OsPROPEP1a	57	10	10	12	10	11	11	11	12	14	15	13	9	8	8	9	8	6	10	7	6	6	5	5	6	15	15	15	11	12	9	14	12	11	13	13	13	11	14	13	17	12	12	14	22	16	56	56	50	49	47	46	50	36	33	43	158		96.86	73.17	29.47	20.72	11.11	11.49	11.05	11.73	10.00	3.57	8.63	5.35	6.18	6.32	7.69	7.44	5.12	5.03
OsPROPEP1b	58	10	10	12	10	11	11	11	12	14	15	13	9	8	8	9	8	6	10	7	6	6	5	5	6	14	14	14	11	12	9	14	12	11	13	13	13	11	14	13	17	12	12	14	22	16	56	56	50	49	47	46	50	36	33	43	153	154		71.34	29.47	19.92	11.11	11.49	11.05	11.73	10.00	3.66	8.85	5.49	6.36	6.45	7.86	7.62	5.24	5.15
ObPROPEP1	59	8	8	11	9	10	10	9	10	12	13	12	7	7	7	6	7	6	8	6	8	8	7	7	8	15	13	13	10	10	8	14	11	13	11	11	11	9	12	13	16	11	10	11	17	12	55	55	45	47	46	44	48	34	32	41	121	120	117		29.03	20.65	10.34	10.17	10.86	10.44	9.29	3.06	8.59	4.79	5.59	6.80	8.62	7.55	5.63	4.06
BdPROPEP1	60	9	10	10	10	12	11	12	13	13	13	12	9	9	9	7	9	6	7	5	7	7	7	7	8	9	9	11	15	15	11	10	10	10	12	12	12	10	12	12	13	14	14	16	15	11	46	49	46	50	41	43	46	36	36	41	57	56	56	54		24.80	12.04	11.92	10.94	7.50	10.00	2.28	7.01	4.29	4.98	8.20	7.44	7.23	4.27	5.07
TuPROPEP1	61	7	7	9	8	7	8	10	8	12	11	9	8	8	7	9	10	8	11	9	8	8	7	7	7	8	9	11	15	14	10	11	10	10	13	13	13	11	10	12	16	11	11	16	15	14	52	52	49	48	49	45	46	38	39	44	53	52	50	51	61		6.72	6.35	5.95	6.32	5.93	2.94	6.72	3.00	3.41	9.27	7.39	4.39	4.07	3.58
GmPROPEP1	62	11	11	13	13	12	10	14	11	13	12	12	10	10	10	10	12	10	10	9	5	5	5	5	7	17	15	15	16	15	14	11	11	22	16	16	17	16	15	16	19	20	14	17	16	14	20	20	14	16	18	20	20	15	15	19	19	19	19	18	23	17		76.67	59.32	31.06	28.03	8.18	15.72	7.80	9.92	2.77	4.19	2.91	9.47	9.27
GmPROPEP2	63	12	12	14	13	12	13	13	14	15	14	13	10	10	10	9	11	9	10	10	6	6	5	5	7	17	17	17	15	15	14	12	12	22	16	16	16	14	15	16	18	19	15	18	17	15	22	22	16	18	21	21	21	14	14	19	20	20	20	18	23	16	92		58.47	37.12	31.82	6.37	13.21	6.21	11.11	3.52	5.05	3.43	10.40	8.97
PvPROPEP9	64	10	10	10	9	9	12	12	14	14	14	10	9	9	9	8	10	8	11	11	5	5	4	4	6	19	18	20	17	17	12	12	12	19	15	15	14	13	13	14	15	18	14	18	16	15	22	22	17	20	18	21	21	14	14	17	19	19	19	19	21	15	70	69		34.09	23.48	7.10	13.38	7.80	8.46	2.78	4.67	3.53	9.52	6.58
MtPROPEP1	65	10	10	10	11	8	10	13	12	14	14	10	7	7	7	10	10	9	10	10	7	7	6	6	6	20	21	20	20	22	16	13	13	18	12	12	12	12	12	12	14	14	12	16	16	13	18	18	15	16	15	14	15	7	8	16	21	21	21	19	15	16	41	49	45		29.55	5.81	11.39	4.73	6.90	2.35	4.61	1.63	9.29	6.06
CaPROPEP9	66	13	13	12	11	14	12	12	9	9	9	13	12	12	12	10	12	11	11	10	7	7	6	6	7	17	17	19	21	20	18	15	13	17	14	14	13	11	15	14	18	17	14	17	20	16	20	20	16	15	19	17	18	11	11	16	18	18	18	17	20	15	37	42	31	39		8.28	12.58	11.81	9.93	2.34	4.57	3.85	6.63	6.17
MnPROPEP5	67	7	7	9	7	7	4	5	3	5	5	6	8	8	8	6	7	10	8	12	7	7	6	6	6	10	12	13	11	10	11	6	7	5	7	7	7	8	7	8	10	8	10	12	11	13	4	4	5	5	8	4	5	7	6	7	7	7	7	6	5	8	13	10	11	9	13		24.46	9.02	11.63	2.16	3.81	3.97	4.46	4.97
PmPROPEP5	68	15	15	14	14	18	13	13	10	13	14	14	9	9	8	8	11	13	15	15	8	8	9	9	8	15	16	17	14	12	15	14	16	15	11	11	10	11	14	16	20	18	12	18	14	14	14	14	15	17	17	13	15	12	11	13	17	17	17	17	15	18	25	21	21	18	20	34		16.30	16.91	4.13	4.39	4.07	9.94	7.36
AtPROPEP5	69	14	14	12	12	14	14	13	13	14	12	17	14	13	15	11	14	12	12	10	8	8	8	9	11	15	13	15	14	12	13	12	11	11	14	14	14	14	14	15	15	13	11	12	7	8	12	12	9	8	11	10	11	11	10	12	10	10	10	9	9	8	11	9	11	7	17	12	22		25.00	2.21	5.24	5.76	8.39	6.61
MtPROPEP2	70	14	14	14	15	17	15	16	16	16	17	17	10	10	10	9	10	10	12	11	8	8	9	9	9	13	11	13	12	10	14	12	11	11	14	14	14	14	16	16	18	17	13	18	14	14	11	11	9	10	12	10	10	10	10	11	11	11	11	10	10	9	13	15	11	10	14	15	23	23		3.64	3.21	6.98	11.94	8.18
ZmPROPEP4	71	5	5	4	6	7	5	4	5	5	6	5	4	4	4	4	6	5	4	5	6	6	7	7	6	8	7	6	4	5	3	4	3	3	9	9	9	8	10	11	12	12	6	6	7	5	19	19	20	22	15	17	18	18	17	23	16	16	16	17	21	24	7	9	7	6	6	5	10	5	8		25.81	6.39	1.49	2.89
ZmPROPEP5	72	6	6	7	9	8	8	7	9	8	9	8	5	6	5	7	6	5	7	8	10	10	9	9	8	7	7	7	9	9	5	5	4	5	8	8	8	8	10	9	12	13	9	6	6	7	25	25	20	22	14	21	22	16	16	21	18	18	18	20	18	19	9	11	10	10	10	8	9	10	6	56		6.87	2.16	4.85
OsPROPEP2	73	6	6	8	8	10	8	10	13	9	11	13	10	10	9	8	11	12	9	7	9	9	10	9	8	8	7	7	7	7	7	7	6	4	11	11	11	9	10	11	9	11	9	9	8	7	18	18	14	15	13	17	17	13	13	15	16	16	16	16	17	13	5	6	6	3	7	6	7	8	9	17	16		7.41	4.00
AcPROPEP	74	12	12	15	13	13	18	22	20	16	18	15	10	10	10	10	11	8	13	10	8	8	8	8	8	22	20	20	20	21	21	16	17	18	20	20	19	18	16	18	12	13	13	15	16	16	12	12	9	9	12	11	11	10	10	12	11	11	11	12	10	12	16	18	16	17	12	7	17	12	16	4	5	8		6.00
GmPROPEP3	75	13	13	15	15	12	13	15	13	9	7	11	12	12	11	12	14	13	12	10	11	11	8	9	9	5	5	5	6	6	6	3	3	9	13	13	12	12	9	10	9	10	11	10	7	8	10	10	8	9	10	11	11	10	10	10	9	10	10	8	11	10	14	14	10	10	10	8	12	8	9	7	10	6	9	

		1	2	3	4	5	6	7	8	9	10	11	12	13	14	15	16	17	18	19	20	21	22	23	24	25	26	27	28	29	30	31	32	33	34	35	36	37	38	39	40	41	42	43	44	45	46	47	48	49	50	51	52	53	54	55	56	57	58	59	60	61	62	63	64	65	66	67	68	69	70	71	72	73	74	75
AaPep7b	1		95.65	86.96	86.96	69.57	30.43	21.74	17.39	21.74	30.43	21.74	33.33	33.33	37.50	29.17	25.00	25.00	37.50	37.50	37.50	33.33	33.33	25.00	25.00	29.17	33.33	33.33	25.00	29.17	29.17	25.00	12.50	12.50	12.50	12.50	12.50	12.50	12.50	16.67	12.50	16.67	16.67	16.67	16.67	16.67	16.67	16.67	16.67	12.50	29.17	29.17	33.33	20.83	29.17	37.50	37.50	12.00	12.00	12.00	16.00	16.00	16.00	11.54	15.38	7.14	16.67	16.67	12.00	16.00	16.00	8.33	12.50	25.00	29.17	12.00
AlPep7	2	22		86.96	86.96	73.91	30.43	21.74	17.39	21.74	30.43	21.74	33.33	33.33	37.50	29.17	25.00	25.00	37.50	37.50	37.50	33.33	33.33	25.00	25.00	33.33	29.17	33.33	29.17	29.17	29.17	25.00	12.50	12.50	12.50	12.50	12.50	12.50	12.50	16.67	12.50	16.67	16.67	16.67	16.67	16.67	16.67	16.67	16.67	12.50	29.17	29.17	33.33	20.83	29.17	37.50	41.67	12.00	12.00	12.00	16.00	16.00	16.00	15.38	15.38	10.71	16.67	16.67	12.00	16.00	16.00	8.33	12.50	25.00	29.17	12.00
AaPep7a	3	20	20		100.00	69.57	30.43	26.09	21.74	26.09	34.78	26.09	37.50	37.50	41.67	29.17	25.00	25.00	33.33	33.33	33.33	29.17	33.33	29.17	33.33	29.17	33.33	37.50	25.00	29.17	25.00	29.17	16.67	16.67	16.67	16.67	16.67	16.67	16.67	20.83	16.67	16.67	16.67	20.83	20.83	20.83	20.83	20.83	20.83	16.67	37.50	37.50	41.67	29.17	37.50	37.50	37.50	12.00	12.00	12.00	16.00	12.00	12.00	11.54	15.38	7.14	16.67	16.67	16.00	20.00	16.00	8.33	16.67	29.17	37.50	12.00
AtPep7	4	20	20	23		69.57	30.43	26.09	21.74	26.09	34.78	26.09	37.50	37.50	41.67	29.17	25.00	25.00	33.33	33.33	33.33	29.17	33.33	29.17	33.33	29.17	33.33	37.50	25.00	29.17	25.00	29.17	16.67	16.67	16.67	16.67	16.67	16.67	16.67	20.83	16.67	16.67	16.67	20.83	20.83	20.83	20.83	20.83	20.83	16.67	37.50	37.50	41.67	29.17	37.50	37.50	37.50	12.00	12.00	12.00	16.00	12.00	12.00	11.54	15.38	7.14	16.67	16.67	16.00	20.00	16.00	8.33	16.67	29.17	37.50	12.00
CrPep7	5	16	17	16	16		26.09	21.74	17.39	21.74	30.43	21.74	37.50	37.50	37.50	33.33	37.50	29.17	33.33	33.33	33.33	33.33	29.17	25.00	20.83	37.50	29.17	25.00	20.83	29.17	29.17	25.00	12.50	12.50	12.50	12.50	16.67	16.67	16.67	12.50	12.50	12.50	12.50	20.83	16.67	16.67	16.67	16.67	12.50	8.33	29.17	33.33	37.50	25.00	33.33	33.33	41.67	20.00	20.00	20.00	20.00	16.00	16.00	15.38	11.54	10.71	16.67	16.67	12.00	16.00	12.00	8.33	12.50	20.83	29.17	16.00
PtPep	6	7	7	7	7	6		52.17	43.48	47.83	43.48	30.43	20.83	20.83	20.83	25.00	29.17	29.17	29.17	29.17	37.50	29.17	37.50	29.17	29.17	25.00	25.00	25.00	29.17	20.83	25.00	25.00	16.67	16.67	16.67	16.67	16.67	20.83	20.83	20.83	16.67	20.83	20.83	20.83	20.83	20.83	20.83	20.83	16.67	16.67	29.17	29.17	29.17	29.17	33.33	37.50	33.33	32.00	32.00	32.00	28.00	24.00	24.00	19.23	19.23	25.00	20.83	41.67	28.00	28.00	28.00	4.17	20.83	41.67	33.33	20.00
RcPep	7	5	5	6	6	5	12		43.48	47.83	60.87	47.83	16.67	16.67	16.67	20.83	25.00	29.17	29.17	29.17	33.33	29.17	33.33	29.17	29.17	25.00	25.00	25.00	25.00	20.83	25.00	29.17	16.67	16.67	16.67	16.67	16.67	16.67	16.67	16.67	20.83	16.67	16.67	16.67	12.50	29.17	29.17	29.17	16.67	12.50	33.33	33.33	33.33	45.83	50.00	41.67	33.33	40.00	40.00	40.00	48.00	36.00	36.00	23.08	23.08	28.57	25.00	25.00	28.00	28.00	28.00	8.33	20.83	50.00	25.00	24.00
GaPep	8	4	4	5	5	4	10	10		69.57	39.13	39.13	25.00	25.00	25.00	29.17	16.67	20.83	20.83	20.83	20.83	16.67	16.67	12.50	12.50	20.83	20.83	8.33	12.50	16.67	20.83	29.17	12.50	12.50	12.50	12.50	12.50	12.50	12.50	12.50	16.67	12.50	12.50	16.67	12.50	20.83	20.83	20.83	12.50	12.50	20.83	20.83	25.00	25.00	25.00	25.00	16.67	40.00	40.00	40.00	24.00	24.00	24.00	19.23	19.23	28.57	20.83	33.33	28.00	28.00	24.00	8.33	12.50	33.33	20.83	24.00
TcPep	9	5	5	6	6	5	11	11	16		43.48	34.78	20.83	20.83	20.83	25.00	20.83	25.00	20.83	20.83	25.00	20.83	25.00	20.83	20.83	20.83	20.83	16.67	16.67	25.00	25.00	37.50	16.67	16.67	16.67	16.67	12.50	12.50	12.50	12.50	12.50	12.50	12.50	16.67	12.50	20.83	20.83	20.83	12.50	12.50	25.00	25.00	25.00	29.17	33.33	29.17	20.83	36.00	36.00	36.00	28.00	28.00	28.00	26.92	23.08	28.57	16.67	37.50	28.00	24.00	32.00	16.67	12.50	33.33	20.83	20.00
CsPep5	10	7	7	8	8	7	10	14	9	10		52.17	20.83	20.83	20.83	25.00	20.83	29.17	25.00	25.00	29.17	29.17	29.17	25.00	29.17	29.17	29.17	25.00	20.83	29.17	33.33	33.33	25.00	25.00	25.00	25.00	25.00	25.00	25.00	20.83	25.00	25.00	25.00	25.00	20.83	33.33	33.33	33.33	25.00	20.83	37.50	37.50	37.50	41.67	45.83	45.83	29.17	32.00	32.00	32.00	32.00	28.00	28.00	38.46	38.46	28.57	29.17	29.17	20.00	20.00	24.00	8.33	29.17	41.67	33.33	24.00
MtPep2	11	5	5	6	6	5	7	11	9	8	12		12.50	12.50	12.50	16.67	12.50	16.67	29.17	29.17	25.00	29.17	29.17	25.00	37.50	33.33	25.00	25.00	25.00	20.83	25.00	25.00	12.50	12.50	12.50	12.50	12.50	12.50	12.50	12.50	12.50	12.50	12.50	16.67	12.50	20.83	20.83	20.83	12.50	20.83	37.50	37.50	37.50	37.50	37.50	33.33	20.83	28.00	28.00	28.00	28.00	20.00	20.00	19.23	19.23	21.43	16.67	20.83	20.00	24.00	20.00	12.50	16.67	37.50	20.83	20.00
AaPep4	12	8	8	9	9	9	5	4	6	5	5	3		100.00	95.65	73.91	58.33	43.48	33.33	33.33	41.67	37.50	29.17	33.33	29.17	20.83	33.33	29.17	12.50	37.50	41.67	20.83	25.00	25.00	25.00	25.00	16.67	20.83	20.83	20.83	25.00	20.83	20.83	16.67	16.67	20.83	20.83	20.83	20.83	12.50	33.33	33.33	33.33	25.00	33.33	25.00	33.33	16.00	16.00	16.00	16.00	16.00	16.00	7.69	8.00	7.14	25.00	16.67	12.00	12.00	8.00	20.83	20.83	16.67	25.00	12.00
AlPep4	13	8	8	9	9	9	5	4	6	5	5	3	23		95.65	73.91	58.33	43.48	33.33	33.33	41.67	37.50	29.17	33.33	29.17	20.83	33.33	29.17	12.50	37.50	41.67	20.83	25.00	25.00	25.00	25.00	16.67	20.83	20.83	20.83	25.00	20.83	20.83	16.67	16.67	20.83	20.83	20.83	20.83	12.50	33.33	33.33	33.33	25.00	33.33	25.00	33.33	16.00	16.00	16.00	16.00	16.00	16.00	7.69	8.00	7.14	25.00	16.67	12.00	12.00	8.00	20.83	20.83	16.67	25.00	12.00
AtPep4	14	9	9	10	10	9	5	4	6	5	5	3	22	22		69.57	58.33	43.48	33.33	33.33	37.50	33.33	29.17	33.33	29.17	20.83	33.33	29.17	12.50	37.50	41.67	20.83	25.00	25.00	25.00	25.00	16.67	20.83	20.83	20.83	25.00	20.83	20.83	16.67	20.83	20.83	20.83	20.83	20.83	16.67	29.17	29.17	29.17	25.00	33.33	25.00	33.33	16.00	16.00	16.00	16.00	16.00	16.00	7.69	8.00	7.14	25.00	12.50	12.00	12.00	8.00	20.83	20.83	16.67	29.17	12.00
EsPep4	15	7	7	7	7	8	6	5	7	6	6	4	17	17	16		45.83	47.83	29.17	29.17	37.50	33.33	29.17	29.17	25.00	16.67	29.17	25.00	12.50	37.50	41.67	20.83	20.83	20.83	20.83	20.83	20.83	20.83	20.83	16.67	20.83	16.67	16.67	16.67	12.50	16.67	16.67	16.67	20.83	12.50	29.17	29.17	29.17	25.00	33.33	25.00	33.33	16.00	16.00	16.00	16.00	16.00	16.00	7.69	8.00	10.71	20.83	20.83	12.00	16.00	8.00	16.67	25.00	20.83	20.83	12.00
CrPep4	16	6	6	6	6	9	7	6	4	5	5	3	14	14	14	11		58.33	33.33	33.33	33.33	33.33	37.50	33.33	29.17	20.83	29.17	29.17	20.83	37.50	37.50	16.67	25.00	25.00	25.00	25.00	16.67	25.00	25.00	20.83	20.83	20.83	20.83	16.67	25.00	25.00	25.00	25.00	20.83	12.50	29.17	29.17	29.17	20.83	29.17	25.00	29.17	24.00	24.00	24.00	16.00	20.00	20.00	7.69	7.69	7.14	16.67	12.50	12.00	12.00	12.00	20.83	20.83	20.83	20.83	12.00
BrPep4	17	6	6	6	6	7	7	7	5	6	7	4	10	10	10	11	14		29.17	29.17	29.17	29.17	33.33	25.00	25.00	25.00	25.00	25.00	16.67	33.33	33.33	16.67	33.33	33.33	33.33	33.33	25.00	33.33	33.33	20.83	25.00	29.17	29.17	25.00	20.83	33.33	33.33	33.33	25.00	20.83	29.17	29.17	29.17	25.00	33.33	25.00	25.00	16.00	16.00	16.00	16.00	16.00	16.00	7.69	8.00	10.71	12.50	20.83	12.00	12.00	8.00	20.83	25.00	25.00	25.00	12.00
AlPep2b	18	9	9	8	8	8	7	7	5	5	6	7	8	8	8	7	8	7		100.00	78.26	69.57	60.87	65.22	56.52	65.22	52.17	43.48	47.83	43.48	43.48	16.67	12.50	12.50	12.50	12.50	12.50	12.50	12.50	12.50	12.50	16.67	16.67	12.50	12.50	25.00	25.00	25.00	12.50	12.50	30.43	30.43	30.43	30.43	30.43	34.78	26.09	20.00	20.00	20.00	20.00	16.00	16.00	8.00	7.69	11.11	20.83	20.83	16.67	16.67	16.00	8.33	12.50	20.83	20.83	16.00
AlPep2c	19	9	9	8	8	8	7	7	5	5	6	7	8	8	8	7	8	7	23		78.26	69.57	60.87	65.22	56.52	65.22	52.17	43.48	47.83	43.48	43.48	16.67	12.50	12.50	12.50	12.50	12.50	12.50	12.50	12.50	12.50	16.67	16.67	12.50	12.50	25.00	25.00	25.00	12.50	12.50	30.43	30.43	30.43	30.43	30.43	34.78	26.09	20.00	20.00	20.00	20.00	16.00	16.00	8.00	7.69	11.11	20.83	20.83	16.67	16.67	16.00	8.33	12.50	20.83	20.83	16.00
AlPep2a	20	9	9	8	8	8	9	8	5	6	7	6	10	10	9	9	8	7	18	18		82.61	65.22	69.57	56.52	56.52	52.17	47.83	43.48	52.17	47.83	16.67	12.50	12.50	12.50	12.50	12.50	12.50	12.50	12.50	12.50	16.67	16.67	12.50	12.50	20.83	20.83	20.83	12.50	8.33	43.48	43.48	43.48	39.13	43.48	34.78	30.43	24.00	24.00	24.00	28.00	24.00	24.00	16.00	11.54	14.81	20.83	20.83	25.00	20.83	20.00	8.33	12.50	20.83	20.83	16.00
AtPep2	21	8	8	7	7	8	7	7	4	5	7	7	9	9	8	8	8	7	16	16	19		65.22	65.22	52.17	56.52	47.83	43.48	43.48	52.17	47.83	16.67	16.67	12.50	12.50	12.50	12.50	12.50	12.50	12.50	12.50	16.67	16.67	16.67	12.50	20.83	20.83	20.83	12.50	12.50	43.48	43.48	43.48	43.48	47.83	34.78	30.43	20.00	20.00	20.00	24.00	24.00	24.00	16.00	11.54	11.11	20.83	20.83	20.83	16.67	16.00	8.33	12.50	25.00	20.83	16.00
AtPep1	22	8	8	8	8	7	9	8	4	6	7	7	7	7	7	7	9	8	14	14	15	15		73.91	69.57	52.17	60.87	65.22	73.91	43.48	47.83	20.83	20.83	20.83	20.83	20.83	16.67	16.67	16.67	20.83	16.67	20.83	20.83	12.50	12.50	29.17	25.00	25.00	20.83	12.50	39.13	39.13	39.13	34.78	39.13	43.48	39.13	28.00	28.00	28.00	32.00	36.00	36.00	20.00	11.54	11.11	16.67	16.67	29.17	25.00	28.00	16.67	16.67	25.00	25.00	20.00
BnPep1	23	6	6	7	7	6	7	7	3	5	6	6	8	8	8	7	8	6	15	15	16	15	17		82.61	47.83	69.57	73.91	60.87	47.83	43.48	12.50	20.83	20.83	20.83	20.83	20.83	20.83	20.83	20.83	20.83	20.83	20.83	16.67	16.67	20.83	20.83	20.83	12.50	16.67	39.13	39.13	39.13	34.78	39.13	30.43	34.78	20.00	20.00	20.00	20.00	24.00	24.00	12.00	7.69	7.41	12.50	12.50	25.00	20.83	20.00	12.50	12.50	20.83	29.17	20.00
EsPep1	24	6	6	8	8	5	7	7	3	5	7	9	7	7	7	6	7	6	13	13	13	12	16	19		43.48	65.22	69.57	65.22	43.48	39.13	12.50	20.83	20.83	20.83	20.83	20.83	20.83	20.83	20.83	20.83	20.83	20.83	16.67	16.67	20.83	20.83	20.83	12.50	16.67	47.83	47.83	47.83	26.09	30.43	30.43	34.78	20.00	20.00	20.00	20.00	20.00	20.00	12.00	7.69	7.41	12.50	12.50	16.67	20.83	20.00	16.67	16.67	20.83	25.00	16.00
CrPep2	25	7	8	7	7	9	6	6	5	5	7	8	5	5	5	4	5	6	15	15	13	13	12	11	10		39.13	34.78	43.48	26.09	26.09	16.67	12.50	12.50	12.50	12.50	20.83	12.50	12.50	16.67	16.67	12.50	12.50	12.50	16.67	20.83	20.83	20.83	12.50	12.50	34.78	39.13	39.13	34.78	34.78	30.43	30.43	20.00	20.00	20.00	20.00	20.00	20.00	12.00	7.69	14.81	20.83	20.83	20.83	16.67	12.00	8.33	16.67	20.83	25.00	16.00
BrPep1a	26	8	7	8	8	7	6	6	5	5	7	6	8	8	8	7	7	6	12	12	12	11	14	16	15	9		60.87	56.52	34.78	34.78	16.67	20.83	20.83	20.83	20.83	20.83	20.83	20.83	20.83	20.83	20.83	20.83	16.67	16.67	20.83	20.83	20.83	12.50	16.67	39.13	39.13	47.83	30.43	34.78	39.13	26.09	16.00	16.00	16.00	20.00	16.00	16.00	12.00	7.69	7.41	12.50	12.50	16.67	16.67	24.00	8.33	12.50	16.67	25.00	12.00
BrPep1b	27	8	8	9	9	6	6	6	2	4	6	6	7	7	7	6	7	6	10	10	11	10	15	17	16	8	14		56.52	34.78	34.78	16.67	20.83	20.83	20.83	20.83	20.83	20.83	20.83	20.83	20.83	20.83	20.83	20.83	20.83	16.67	16.67	16.67	12.50	20.83	43.48	43.48	43.48	34.78	39.13	39.13	34.78	16.00	16.00	16.00	20.00	24.00	24.00	16.00	7.69	3.70	8.33	8.33	16.67	12.50	20.00	16.67	16.67	16.67	20.83	12.00
CrPep1	28	6	7	6	6	5	7	6	3	4	5	6	3	3	3	3	5	4	11	11	10	10	17	14	15	10	13	13		26.09	34.78	12.50	25.00	25.00	25.00	25.00	20.83	20.83	20.83	25.00	20.83	25.00	25.00	16.67	16.67	25.00	20.83	20.83	16.67	16.67	34.78	34.78	30.43	21.74	21.74	39.13	34.78	24.00	24.00	24.00	20.00	20.00	20.00	16.00	3.85	11.11	12.50	12.50	20.83	20.83	32.00	8.33	12.50	20.83	20.83	12.00
AtPep8	29	7	7	7	7	7	5	5	4	6	7	5	9	9	9	9	9	8	10	10	12	12	10	11	10	6	8	8	6		73.91	20.83	16.67	12.50	12.50	12.50	12.50	12.50	12.50	16.67	12.50	12.50	12.50	16.67	12.50	20.83	20.83	20.83	16.67	8.33	34.78	34.78	34.78	34.78	43.48	30.43	34.78	20.00	20.00	20.00	16.00	20.00	20.00	20.00	15.38	11.11	16.67	12.50	12.50	12.50	12.00	12.50	12.50	25.00	20.83	16.00
EsPep8	30	7	7	6	6	7	6	6	5	6	8	6	10	10	10	10	9	8	10	10	11	11	11	10	9	6	8	8	8	17		25.00	20.83	16.67	16.67	16.67	12.50	16.67	16.67	12.50	12.50	20.83	20.83	16.67	16.67	25.00	20.83	20.83	16.67	12.50	34.78	34.78	30.43	34.78	43.48	39.13	43.48	24.00	24.00	24.00	20.00	20.00	20.00	16.00	11.54	14.81	16.67	12.50	16.67	16.67	24.00	12.50	12.50	20.83	25.00	20.00
CaPep9	31	6	6	7	7	6	6	7	7	9	8	6	5	5	5	5	4	4	4	4	4	4	5	3	3	4	4	4	3	5	6		20.83	20.83	20.83	20.83	16.67	20.83	20.83	12.50	16.67	16.67	16.67	20.83	20.83	25.00	20.83	20.83	16.67	12.50	20.83	20.83	20.83	25.00	29.17	25.00	33.33	29.17	29.17	29.17	29.17	37.50	37.50	30.77	26.92	25.93	25.00	16.67	36.00	36.00	32.00	8.33	16.67	20.83	33.33	32.00
ObPep1	32	3	3	4	4	3	4	4	3	4	6	3	6	6	6	5	6	8	3	3	3	4	5	5	5	3	5	5	6	4	5	5		95.65	95.65	95.65	73.91	70.83	70.83	56.52	65.22	73.91	73.91	54.17	54.17	60.87	56.52	56.52	65.22	47.83	20.83	20.83	20.83	29.17	29.17	20.83	16.67	16.00	16.00	16.00	12.00	12.00	12.00	0.00	4.00	7.14	20.83	16.67	28.00	28.00	20.83	20.83	50.00	30.43	25.00	12.00
OsiPep1	33	3	3	4	4	3	4	4	3	4	6	3	6	6	6	5	6	8	3	3	3	3	5	5	5	3	5	5	6	3	4	5	22		100.00	100.00	73.91	75.00	75.00	60.87	69.57	73.91	73.91	54.17	58.33	60.87	60.87	60.87	69.57	52.17	20.83	20.83	20.83	25.00	25.00	20.83	16.67	16.00	16.00	16.00	12.00	12.00	12.00	0.00	4.00	7.14	20.83	16.67	28.00	28.00	20.83	20.83	54.17	26.09	25.00	12.00
OsPep1a	34	3	3	4	4	3	4	4	3	4	6	3	6	6	6	5	6	8	3	3	3	3	5	5	5	3	5	5	6	3	4	5	22	23		100.00	73.91	75.00	75.00	60.87	69.57	73.91	73.91	54.17	58.33	60.87	60.87	60.87	69.57	52.17	20.83	20.83	20.83	25.00	25.00	20.83	16.67	16.00	16.00	16.00	12.00	12.00	12.00	0.00	4.00	7.14	20.83	16.67	28.00	28.00	20.83	20.83	54.17	26.09	25.00	12.00
OsPep1b	35	3	3	4	4	3	4	4	3	4	6	3	6	6	6	5	6	8	3	3	3	3	5	5	5	3	5	5	6	3	4	5	22	23	23		73.91	75.00	75.00	60.87	69.57	73.91	73.91	54.17	58.33	60.87	60.87	60.87	69.57	52.17	20.83	20.83	20.83	25.00	25.00	20.83	16.67	16.00	16.00	16.00	12.00	12.00	12.00	0.00	4.00	7.14	20.83	16.67	28.00	28.00	20.83	20.83	54.17	26.09	25.00	12.00
BdPep1	36	3	3	4	4	4	4	4	3	3	6	3	4	4	4	5	4	6	3	3	3	3	4	5	5	5	5	5	5	3	3	4	17	17	17	17		66.67	66.67	52.17	69.57	65.22	65.22	50.00	54.17	52.17	60.87	60.87	52.17	43.48	20.83	25.00	25.00	25.00	25.00	20.83	12.50	20.00	20.00	20.00	12.00	12.00	12.00	0.00	4.00	3.57	20.83	16.67	16.00	20.00	16.67	16.67	50.00	26.09	25.00	12.00
SiPep1a	37	3	3	4	4	4	5	4	3	3	6	3	5	5	5	5	6	8	3	3	3	3	4	5	5	3	5	5	5	3	4	5	17	18	18	18	16		100.00	58.33	66.67	75.00	75.00	58.33	58.33	54.17	58.33	58.33	58.33	50.00	20.83	20.83	20.83	20.83	20.83	20.83	20.83	12.00	12.00	12.00	8.00	8.00	8.00	0.00	3.85	7.41	25.00	20.83	24.00	24.00	20.00	25.00	62.50	29.17	29.17	16.00
SiPep1b	38	3	3	4	4	4	5	4	3	3	6	3	5	5	5	5	6	8	3	3	3	3	4	5	5	3	5	5	5	3	4	5	17	18	18	18	16	23		58.33	66.67	75.00	75.00	58.33	58.33	54.17	58.33	58.33	58.33	50.00	20.83	20.83	20.83	20.83	20.83	20.83	20.83	12.00	12.00	12.00	8.00	8.00	8.00	0.00	3.85	7.41	25.00	20.83	24.00	24.00	20.00	25.00	62.50	29.17	29.17	16.00
SbPep2	39	4	4	5	5	3	5	4	3	3	5	3	5	5	5	4	5	5	3	3	3	3	5	5	5	4	5	5	6	4	3	3	13	14	14	14	12	14	14		73.91	56.52	56.52	45.83	50.00	56.52	47.83	47.83	65.22	52.17	20.83	20.83	20.83	16.67	16.67	20.83	12.50	16.00	16.00	16.00	16.00	12.00	12.00	0.00	4.00	3.57	20.83	12.50	20.00	20.00	16.67	20.83	45.83	30.43	20.83	12.00
ZmPep2	40	3	3	4	4	3	4	5	4	3	6	3	6	6	6	5	5	6	3	3	3	3	4	5	5	4	5	5	5	3	3	4	15	16	16	16	16	16	16	17		65.22	65.22	45.83	54.17	47.83	56.52	56.52	56.52	47.83	20.83	20.83	20.83	25.00	25.00	20.83	12.50	16.00	16.00	16.00	12.00	12.00	12.00	0.00	4.00	3.57	20.83	12.50	16.00	16.00	16.67	16.67	45.83	26.09	20.83	12.00
ZmPep1b	41	4	4	4	4	3	5	4	3	3	6	3	5	5	5	4	5	7	4	4	4	4	5	5	5	3	5	5	6	3	5	4	17	17	17	17	15	18	18	13	15		100.00	58.33	62.50	65.22	56.52	56.52	60.87	52.17	20.83	20.83	20.83	20.83	20.83	25.00	16.67	12.00	12.00	12.00	12.00	8.00	8.00	0.00	8.00	7.14	29.17	16.67	24.00	20.00	25.00	33.33	54.17	30.43	25.00	12.00
ZmPep1	42	4	4	4	4	3	5	4	3	3	6	3	5	5	5	4	5	7	4	4	4	4	5	5	5	3	5	5	6	3	5	4	17	17	17	17	15	18	18	13	15	23		58.33	62.50	65.22	56.52	56.52	60.87	52.17	20.83	20.83	20.83	20.83	20.83	25.00	16.67	12.00	12.00	12.00	12.00	8.00	8.00	0.00	8.00	7.14	29.17	16.67	24.00	20.00	25.00	33.33	54.17	30.43	25.00	12.00
OsPep2	43	4	4	5	5	5	5	4	4	4	6	4	4	4	4	4	4	6	3	3	3	4	3	4	4	3	4	5	4	4	4	5	13	13	13	13	12	14	14	11	11	14	14		65.22	50.00	45.83	45.83	41.67	54.17	16.67	16.67	16.67	20.83	20.83	16.67	20.83	8.00	8.00	8.00	4.00	4.00	4.00	3.85	7.69	14.29	25.00	20.83	16.00	24.00	24.00	12.50	33.33	29.17	20.83	12.00
ZmPep5	44	4	4	5	5	4	5	3	3	3	5	3	4	4	5	3	6	5	3	3	3	3	3	4	4	4	4	5	4	3	4	5	13	14	14	14	13	14	14	12	13	15	15	15		54.17	50.00	50.00	50.00	62.50	16.67	16.67	16.67	20.83	20.83	16.67	16.67	12.00	12.00	12.00	8.00	4.00	4.00	0.00	3.85	7.14	25.00	16.67	16.00	16.00	16.00	16.67	41.67	25.00	25.00	8.00
SbPep1	45	4	4	5	5	4	5	7	5	5	8	5	5	5	5	4	6	8	6	6	5	5	7	5	5	5	5	4	6	5	6	6	14	14	14	14	12	13	13	13	11	15	15	12	13		73.91	73.91	65.22	52.17	25.00	25.00	25.00	25.00	25.00	29.17	20.83	20.00	20.00	20.00	20.00	16.00	16.00	8.00	12.00	14.29	33.33	20.83	28.00	28.00	20.83	25.00	50.00	34.78	29.17	20.00
ZmPep1a	46	4	4	5	5	4	5	7	5	5	8	5	5	5	5	4	6	8	6	6	5	5	6	5	5	5	5	4	5	5	5	5	13	14	14	14	14	14	14	11	13	13	13	11	12	17		100.00	56.52	47.83	25.00	25.00	25.00	25.00	25.00	29.17	16.67	20.00	20.00	20.00	12.00	12.00	12.00	8.00	8.00	10.71	25.00	20.83	20.00	20.00	16.67	20.83	50.00	34.78	25.00	16.00
ZmPep3	47	4	4	5	5	4	5	7	5	5	8	5	5	5	5	4	6	8	6	6	5	5	6	5	5	5	5	4	5	5	5	5	13	14	14	14	14	14	14	11	13	13	13	11	12	17	23		56.52	47.83	25.00	25.00	25.00	25.00	25.00	29.17	16.67	20.00	20.00	20.00	12.00	12.00	12.00	8.00	8.00	10.71	25.00	20.83	20.00	20.00	16.67	20.83	50.00	34.78	25.00	16.00
TuPep1	48	4	4	5	5	3	4	4	3	3	6	3	5	5	5	5	5	6	3	3	3	3	5	3	3	3	3	3	4	4	4	4	15	16	16	16	12	14	14	15	13	14	14	10	12	15	13	13		56.52	20.83	20.83	20.83	16.67	16.67	20.83	16.67	20.00	20.00	20.00	24.00	20.00	20.00	8.00	16.00	7.14	29.17	25.00	24.00	28.00	16.67	29.17	58.33	34.78	25.00	20.00
ZmPep4	49	3	3	4	4	2	4	3	3	3	5	5	3	3	4	3	3	5	3	3	2	3	3	4	4	3	4	5	4	2	3	3	11	12	12	12	10	12	12	12	11	12	12	13	15	15	11	11	13		12.50	12.50	12.50	16.67	16.67	12.50	12.50	4.00	4.00	4.00	8.00	4.00	4.00	0.00	0.00	10.71	16.67	20.83	12.00	12.00	16.67	20.83	41.67	26.09	20.83	12.00
AaPep3	50	7	7	9	9	7	7	8	5	6	9	9	8	8	7	7	7	7	7	7	10	10	9	9	11	8	9	10	8	8	8	5	5	5	5	5	5	5	5	5	5	5	5	4	4	6	6	6	5	3		95.65	86.96	47.83	56.52	52.17	34.78	16.00	16.00	16.00	20.00	16.00	16.00	16.00	11.54	14.81	12.50	20.83	20.83	20.83	20.00	16.67	29.17	25.00	33.33	12.00
AtPep3	51	7	7	9	9	8	7	8	5	6	9	9	8	8	7	7	7	7	7	7	10	10	9	9	11	9	9	10	8	8	8	5	5	5	5	5	5	6	5	5	5	5	5	4	4	6	6	6	5	3	22		91.30	47.83	56.52	52.17	34.78	20.00	20.00	20.00	24.00	20.00	20.00	16.00	11.54	14.81	12.50	20.83	20.83	20.83	20.00	16.67	29.17	25.00	37.50	16.00
AlPep3	52	8	8	10	10	9	7	8	6	6	9	9	8	8	7	7	7	7	7	7	10	10	9	9	11	9	11	10	7	8	7	5	5	5	5	5	5	6	5	5	5	5	5	4	4	6	6	6	5	3	20	21		47.83	56.52	52.17	34.78	24.00	24.00	24.00	24.00	20.00	20.00	16.00	11.54	14.81	12.50	20.83	20.83	20.83	16.00	16.67	29.17	25.00	37.50	16.00
BrPep3	53	5	5	7	7	6	7	11	6	7	10	9	6	6	6	6	5	6	7	7	9	10	8	8	6	8	7	8	5	8	8	6	7	6	6	6	6	5	5	4	6	5	5	5	5	6	6	6	4	4	11	11	11		91.30	43.48	26.09	20.00	20.00	20.00	24.00	20.00	20.00	12.00	7.69	14.81	16.67	20.83	25.00	16.67	12.00	12.50	29.17	37.50	25.00	8.00
EsPep3	54	7	7	9	9	8	8	12	6	8	11	9	8	8	8	8	7	8	7	7	10	11	9	9	7	8	8	9	5	10	10	7	7	6	6	6	6	5	5	4	6	5	5	5	5	6	6	6	4	4	13	13	13	21		52.17	34.78	20.00	20.00	20.00	28.00	24.00	24.00	16.00	11.54	14.81	16.67	20.83	25.00	16.67	12.00	12.50	29.17	37.50	29.17	8.00
CrPep3	55	9	9	9	9	8	9	10	6	7	11	8	6	6	6	6	6	6	8	8	8	8	10	7	7	7	9	9	9	7	9	6	5	5	5	5	5	5	5	5	5	6	6	4	4	7	7	7	5	3	12	12	12	10	12		39.13	20.00	20.00	20.00	32.00	20.00	20.00	24.00	19.23	14.81	12.50	20.83	20.83	20.83	28.00	12.50	25.00	33.33	33.33	16.00
AtPep5	56	9	10	9	9	10	8	8	4	5	7	5	8	8	8	8	7	6	6	6	7	7	9	8	8	7	6	8	8	8	10	8	4	4	4	4	3	5	5	3	3	4	4	5	4	5	4	4	4	3	8	8	8	6	8	9		16.00	16.00	16.00	20.00	16.00	16.00	24.00	15.38	22.22	29.17	20.83	20.83	25.00	24.00	8.33	16.67	25.00	33.33	20.00
NbPep6	57	3	3	3	3	5	8	10	10	9	8	7	4	4	4	4	6	4	5	5	6	5	7	5	5	5	4	4	6	5	6	7	4	4	4	4	5	3	3	4	4	3	3	2	3	5	5	5	5	1	4	5	6	5	5	5	4		100.00	100.00	65.22	65.22	65.22	32.00	36.00	33.33	24.00	20.00	33.33	33.33	29.17	8.33	8.00	32.00	20.00	37.50
NsPep6	58	3	3	3	3	5	8	10	10	9	8	7	4	4	4	4	6	4	5	5	6	5	7	5	5	5	4	4	6	5	6	7	4	4	4	4	5	3	3	4	4	3	3	2	3	5	5	5	5	1	4	5	6	5	5	5	4	23		100.00	65.22	65.22	65.22	32.00	36.00	33.33	24.00	20.00	33.33	33.33	29.17	8.33	8.00	32.00	20.00	37.50
NtPep6	59	3	3	3	3	5	8	10	10	9	8	7	4	4	4	4	6	4	5	5	6	5	7	5	5	5	4	4	6	5	6	7	4	4	4	4	5	3	3	4	4	3	3	2	3	5	5	5	5	1	4	5	6	5	5	5	4	23	23		65.22	65.22	65.22	32.00	36.00	33.33	24.00	20.00	33.33	33.33	29.17	8.33	8.00	32.00	20.00	37.50
SmPep1	60	4	4	4	4	5	7	12	6	7	8	7	4	4	4	4	4	4	5	5	7	6	8	5	5	5	5	5	5	4	5	7	3	3	3	3	3	2	2	4	3	3	3	1	2	5	3	3	6	2	5	6	6	6	7	8	5	15	15	15		73.91	73.91	40.00	40.00	33.33	28.00	20.00	33.33	33.33	20.83	16.67	16.00	32.00	24.00	37.50
SlPep6	61	4	4	3	3	4	6	9	6	7	7	5	4	4	4	4	5	4	4	4	6	6	9	6	5	5	4	6	5	5	5	9	3	3	3	3	3	2	2	3	3	2	2	1	1	4	3	3	5	1	4	5	5	5	6	5	4	15	15	15	17		95.65	40.00	40.00	25.93	24.00	20.00	29.17	25.00	25.00	16.67	12.00	32.00	16.00	41.67
StPep1	62	4	4	3	3	4	6	9	6	7	7	5	4	4	4	4	5	4	4	4	6	6	9	6	5	5	4	6	5	5	5	9	3	3	3	3	3	2	2	3	3	2	2	1	1	4	3	3	5	1	4	5	5	5	6	5	4	15	15	15	17	22		40.00	40.00	25.93	24.00	20.00	29.17	25.00	25.00	16.67	12.00	32.00	16.00	41.67
AtPep6	63	3	4	3	3	4	5	6	5	7	10	5	2	2	2	2	2	2	2	2	4	4	5	3	3	3	3	4	4	5	4	8	0	0	0	0	0	0	0	0	0	0	0	1	0	2	2	2	2	0	4	4	4	3	4	6	6	8	8	8	10	10	10		75.00	40.74	26.92	19.23	16.67	20.83	20.83	8.00	3.85	20.00	11.54	24.00
BrPep6	64	4	4	4	4	3	5	6	5	6	10	5	2	2	2	2	2	2	2	2	3	3	3	2	2	2	2	2	1	4	3	7	1	1	1	1	1	1	1	1	1	2	2	2	1	3	2	2	4	0	3	3	3	2	3	5	4	9	9	9	10	10	10	18		35.71	34.62	19.23	16.00	20.00	16.67	4.00	3.85	24.00	11.54	24.00
VvPep6	65	2	3	2	2	3	7	8	8	8	8	6	2	2	2	3	2	3	3	3	4	3	3	2	2	4	2	1	3	3	4	7	2	2	2	2	1	2	2	1	1	2	2	4	2	4	3	3	2	3	4	4	4	4	4	4	6	9	9	9	9	7	7	11	10		21.43	17.86	29.63	33.33	25.00	3.57	3.70	25.00	21.43	28.57
MnPep5	66	4	4	4	4	4	5	6	5	4	7	4	6	6	6	5	4	3	5	5	5	5	4	3	3	5	3	2	3	4	4	6	5	5	5	5	5	6	6	5	5	7	7	6	6	8	6	6	7	4	3	3	3	4	4	3	7	6	6	6	7	6	6	7	9	6		34.78	24.00	28.00	16.00	16.67	29.17	29.17	20.83	16.00
PmPep5	67	4	4	4	4	4	10	6	8	9	7	5	4	4	3	5	3	5	5	5	5	5	4	3	3	5	3	2	3	3	3	4	4	4	4	4	4	5	5	3	3	4	4	5	4	5	5	5	6	5	5	5	5	5	5	5	5	5	5	5	5	5	5	5	5	5	8		20.00	20.00	12.00	16.67	25.00	33.33	20.83	24.00
GmPep1	68	3	3	4	4	3	7	7	7	7	5	5	3	3	3	3	3	3	4	4	6	5	7	6	4	5	4	4	5	3	4	9	7	7	7	7	4	6	6	5	4	6	6	4	4	7	5	5	6	3	5	5	5	6	6	5	5	8	8	8	8	7	7	4	4	8	6	5		78.26	37.50	12.50	20.00	20.00	48.00	37.50
GmPep2	69	4	4	5	5	4	7	7	7	6	5	6	3	3	3	4	3	3	4	4	5	4	6	5	5	4	4	3	5	3	4	9	7	7	7	7	5	6	6	5	4	5	5	6	4	7	5	5	7	3	5	5	5	4	4	5	6	8	8	8	8	6	6	5	5	9	7	5	18		50.00	8.33	24.00	20.00	48.00	41.67
PvPep9	70	4	4	4	4	3	7	6	6	8	6	5	2	2	2	2	3	2	4	4	5	4	7	5	5	3	6	5	8	3	6	8	5	5	5	5	4	5	5	4	4	6	6	6	4	5	4	4	4	4	5	5	4	3	3	7	6	7	7	7	5	6	6	5	4	7	4	3	9	12		8.33	12.00	20.83	28.00	29.17
AcPep	71	2	2	2	2	2	1	2	2	4	2	3	5	5	5	4	5	5	2	2	2	2	4	3	4	2	2	4	2	3	3	2	5	5	5	5	4	6	6	5	4	8	8	3	4	6	5	5	7	5	4	4	4	3	3	3	2	2	2	2	4	4	4	2	1	1	4	4	3	2	2		45.83	25.00	8.33	20.83
SbPep3	72	3	3	4	4	3	5	5	3	3	7	4	5	5	5	6	5	6	3	3	3	3	4	3	4	4	3	4	3	3	3	4	12	13	13	13	12	15	15	11	11	13	13	8	10	12	12	12	14	10	7	7	7	7	7	6	4	2	2	2	4	3	3	1	1	1	7	6	5	6	3	11		33.33	25.00	16.00
RcPep5	73	6	6	7	7	5	10	12	8	8	10	9	4	4	4	5	5	6	5	5	5	6	6	5	5	5	4	4	5	6	5	5	7	6	6	6	6	7	7	7	6	7	7	7	6	8	8	8	8	6	6	6	6	9	9	8	6	8	8	8	8	8	8	5	6	7	7	8	5	5	5	6	8		33.33	24.00
GmPep3	74	7	7	9	9	7	8	6	5	5	8	5	6	6	7	5	5	6	5	5	5	5	6	7	6	6	6	5	5	5	6	8	6	6	6	6	6	7	7	5	5	6	6	5	6	7	6	6	6	5	8	9	9	6	7	8	5	5	5	5	6	4	4	3	3	6	5	5	12	12	7	2	6	8		41.67
MtPep1	75	3	3	3	3	4	5	6	6	5	6	5	3	3	3	3	3	3	4	4	4	4	5	5	4	4	3	3	3	4	5	8	3	3	3	3	3	4	4	3	3	3	3	3	2	5	4	4	5	3	3	4	4	2	2	4	5	9	9	9	9	10	10	6	6	8	4	6	9	10	7	5	4	6	10	

		1	2	3	4	5	6	7	8	9	10	11	12	13	14	15	16	17	18	19	20	21	22	23	24	25	26	27	28	29	30	31	32	33	34	35	36	37	38	39	40	41	42
PtPEPR1a	1		69.80	65.32	61.75	63.04	59.86	60.07	55.71	54.12	54.43	54.02	57.21	55.11	54.99	56.00	54.87	51.37	54.94	53.16	53.33	53.83	53.85	44.35	52.17	52.43	51.87	42.02	50.74	50.96	47.70	47.41	45.46	45.56	46.45	46.10	46.21	46.05	47.91	42.64	44.91	41.47	46.61
PtPEPR1b	2	779		60.16	54.91	59.65	57.42	59.38	51.47	50.49	50.63	50.27	53.87	52.58	53.45	50.93	50.00	47.01	53.19	49.91	50.00	50.09	51.45	41.95	48.48	48.39	48.12	43.34	46.82	46.87	48.97	48.71	42.62	42.84	43.53	43.73	43.17	43.37	44.09	44.02	39.78	41.22	43.32
CsPEPR1	3	729	669		58.19	63.73	60.45	62.19	55.43	54.45	54.85	54.62	57.37	55.18	55.14	56.38	54.81	51.56	55.48	54.45	54.80	55.09	53.20	45.29	52.43	52.52	51.96	41.97	48.99	49.39	45.71	48.76	45.93	46.54	47.01	47.31	47.56	47.74	47.69	44.08	44.95	42.10	46.30
LuPEPR1	4	707	626	664		55.83	54.16	54.02	51.40	50.09	51.53	50.48	51.88	51.53	48.57	52.48	51.04	47.43	51.52	50.17	50.52	49.17	49.00	42.60	50.22	50.65	49.96	40.29	47.40	47.71	44.26	44.53	45.11	45.93	45.55	45.58	45.86	45.99	46.94	41.98	44.13	40.49	44.74
PpPEPR1a	5	706	652	710	637		84.83	76.95	57.35	55.21	55.61	55.29	58.65	56.56	57.18	55.90	54.86	51.69	55.74	53.35	53.35	53.46	53.78	46.33	51.30	51.22	50.52	42.55	48.81	49.29	46.66	49.50	45.53	45.97	45.88	45.52	45.89	46.21	47.07	45.34	43.61	42.53	45.77
PpPEPR1b	6	674	631	677	618	928		73.18	56.64	54.87	55.00	55.04	56.70	54.58	56.02	53.36	52.42	50.71	54.35	52.46	52.46	52.48	52.78	45.96	48.53	48.40	48.01	39.68	47.36	47.85	45.73	48.83	45.10	45.19	45.19	45.08	45.15	45.73	46.25	43.09	43.18	42.10	44.55
MdPEPR1	7	674	636	694	618	838	802		57.08	55.21	55.16	55.11	57.70	56.38	55.62	54.39	53.89	50.89	55.24	52.47	52.47	52.34	52.46	45.93	50.70	50.00	50.61	42.91	48.98	49.82	48.67	51.74	45.15	45.58	46.01	45.39	46.90	46.69	47.77	45.37	43.65	43.76	45.82
GmPEPR1a	8	629	576	623	588	644	640	641		87.72	81.94	71.19	62.08	59.50	49.83	53.85	53.70	55.46	52.55	50.98	51.34	50.99	51.47	41.91	49.38	50.09	49.34	39.52	47.48	48.05	44.95	46.52	45.22	44.48	45.26	44.65	44.82	44.32	45.35	41.16	42.99	39.79	44.58
GmPEPR2	9	611	565	612	573	620	620	620	950		79.74	70.28	61.82	59.02	49.08	52.42	52.00	54.28	50.94	50.81	50.89	50.09	50.04	42.71	48.06	48.94	47.92	39.73	46.32	46.37	44.40	45.09	44.17	43.87	44.56	43.95	44.21	44.14	44.47	40.46	42.65	39.54	44.14
PvPEPR1b	10	614	566	616	588	624	621	620	894	870		70.16	61.51	59.86	48.91	51.91	51.95	54.12	51.33	50.36	50.71	49.82	51.33	42.42	48.02	48.90	48.59	38.97	46.87	47.09	44.25	45.59	44.43	43.79	44.31	44.56	44.31	44.06	44.44	40.81	42.94	39.91	44.62
MtPEPR1	11	611	563	615	578	622	623	620	776	766	769		59.46	57.03	49.13	51.78	51.91	53.58	52.00	49.07	49.15	48.39	48.71	41.50	47.28	48.24	47.67	38.22	45.59	45.99	43.49	45.67	42.92	42.62	42.92	42.83	43.78	43.41	43.96	39.60	41.63	38.44	43.54
GmPEPR1b	12	647	606	646	594	661	643	652	691	680	684	666		79.65	51.58	53.76	54.14	53.40	53.19	51.50	51.86	51.51	51.54	46.13	48.43	48.69	48.12	41.30	46.71	46.67	43.73	46.68	44.53	44.41	44.70	44.95	45.31	45.11	45.79	41.31	42.24	38.72	43.37
PvPEPR1a	13	625	592	623	591	638	620	636	661	648	665	637	869		50.00	53.19	53.83	52.06	52.21	50.31	51.02	50.04	52.11	46.49	47.86	48.30	47.99	40.76	45.44	45.57	42.59	45.89	44.47	44.26	44.35	44.77	45.57	45.14	45.83	41.12	41.83	38.82	44.26
VvPEPR1	14	634	589	633	562	641	628	614	571	560	560	564	587	567		51.00	49.70	45.16	51.57	51.74	51.39	51.57	50.96	47.64	44.00	44.60	44.85	43.47	45.00	44.74	45.40	51.87	42.51	42.63	42.94	43.28	42.29	42.25	43.33	45.72	40.94	42.09	42.37
CsaPEPR1a	15	635	575	636	604	630	604	613	602	586	584	581	607	601	588		79.93	49.87	52.22	51.25	51.42	51.02	51.24	42.55	49.78	49.83	50.09	40.48	47.50	48.07	44.23	45.42	43.41	43.93	43.59	43.97	44.23	43.99	44.76	41.37	41.78	39.11	43.69
CsaPEPR1b	16	625	567	621	590	621	596	610	603	584	587	585	614	611	572	888		50.36	51.64	50.22	51.02	50.13	50.79	42.57	48.17	48.13	48.65	39.75	46.42	47.20	43.71	45.47	43.15	43.66	43.58	43.49	44.20	43.87	44.63	41.17	42.49	39.91	43.80
PvPEPR1c	17	581	527	580	544	581	573	572	609	596	598	591	597	582	518	557	565		49.06	47.54	48.08	47.70	47.77	40.64	45.28	45.06	45.49	34.91	42.88	43.64	41.09	45.13	41.50	41.33	41.45	41.62	42.13	41.98	42.48	38.36	39.53	36.25	40.28
AcPEPR1	18	623	601	628	595	631	618	627	588	570	577	584	601	591	593	589	582	549		51.50	52.38	53.36	52.85	46.37	49.00	48.87	49.56	42.37	46.88	46.97	43.89	51.57	45.96	46.83	47.30	47.08	47.29	46.61	46.64	43.30	43.59	41.86	46.13
SlPEPR1	19	606	565	618	575	605	596	595	570	568	565	551	583	570	596	576	568	531	584		94.75	79.69	64.66	49.96	48.99	48.86	48.90	38.74	45.92	45.88	42.76	44.34	44.26	45.43	45.82	46.15	44.88	44.89	45.66	42.56	43.11	40.34	45.45
StPEPR1	20	608	566	622	579	605	596	595	574	569	569	552	587	578	592	578	577	537	594	1046		80.60	65.02	49.42	49.17	49.21	49.34	38.92	46.44	46.23	43.02	44.42	44.35	45.52	45.56	46.07	45.31	45.23	45.40	42.47	43.28	40.77	45.62
NbPEPR1	21	611	563	622	562	603	593	592	567	557	555	540	580	564	590	573	566	530	595	883	893		63.61	51.29	48.73	48.07	48.24	40.55	45.64	46.04	43.48	45.88	44.69	45.52	45.38	46.60	46.10	45.58	45.27	42.62	43.44	40.55	45.27
InPEPR1	22	616	584	606	561	612	599	597	577	561	577	548	586	592	585	579	577	535	602	721	725	708		49.56	48.69	49.00	49.30	39.30	45.68	45.81	42.57	44.74	45.98	46.46	46.37	46.37	45.79	45.74	46.83	43.22	44.47	41.70	45.44
EgPEPR1	23	506	469	514	489	517	512	514	469	469	476	466	512	516	514	480	481	454	518	560	554	558	557		40.35	40.75	40.65	44.19	40.41	41.14	40.00	43.86	38.52	39.51	39.21	39.35	39.60	39.74	39.77	40.73	37.28	35.90	38.86
AlPEPR1	24	600	557	603	582	590	561	583	560	545	547	538	555	548	513	569	553	513	561	559	561	555	558	462		92.97	85.93	64.15	63.50	64.51	59.43	42.93	44.43	44.62	44.66	45.91	44.88	45.01	45.75	41.65	42.15	39.39	43.17
AtPEPR1	25	603	556	604	587	589	560	575	568	555	557	549	558	553	520	570	553	511	560	558	562	548	562	467	1044		84.34	63.56	63.77	64.42	59.82	43.07	44.95	44.96	45.17	46.25	45.05	45.36	45.92	41.83	41.81	39.22	42.91
EsPEPR1	26	595	551	596	579	580	554	581	558	542	552	541	550	548	523	571	557	514	566	558	563	549	565	465	965	948		65.81	63.29	64.30	59.82	43.00	44.81	45.16	44.77	45.68	45.56	45.74	46.17	41.88	42.51	39.59	43.92
BrPEPR1	27	479	462	478	465	474	444	469	443	437	438	430	458	452	459	457	450	392	472	437	439	444	446	426	723	717	741		51.59	51.81	54.70	42.93	35.37	35.75	35.46	36.09	36.03	36.52	36.61	39.33	33.45	36.15	34.79
AlPEPR2	28	580	530	560	547	552	539	555	536	523	531	517	532	518	513	542	531	485	534	523	529	518	523	451	715	718	712	569		91.54	78.75	43.58	41.77	42.13	41.82	42.82	43.28	43.12	43.63	41.57	41.30	39.48	41.65
AtPEPR2	29	583	531	565	551	558	545	565	543	524	534	522	532	520	510	549	540	494	535	523	527	523	525	460	727	726	724	572	996		79.43	44.12	41.85	42.13	41.73	42.56	42.84	43.03	43.63	42.02	41.70	39.97	41.88
CrPEPR2	30	540	522	517	509	517	509	529	498	492	493	484	492	480	493	498	493	454	492	481	484	480	481	426	668	673	670	553	856	865		44.78	38.77	39.58	39.54	39.65	39.73	39.59	40.71	42.61	38.56	40.52	39.12
AcPEPR2	31	539	527	552	513	549	544	565	521	505	512	512	528	519	555	511	512	505	575	501	502	507	506	461	489	491	488	434	485	491	463		41.54	41.63	42.28	42.24	43.21	42.96	42.29	46.35	40.31	41.75	41.65
ZmPEPR1a	32	526	491	531	526	525	520	521	520	508	511	494	517	515	491	501	498	476	529	513	514	513	532	441	515	521	518	405	482	483	442	474		88.54	89.16	80.99	72.13	71.68	72.69	60.18	61.50	57.40	62.61
ZmPEPR1b	33	528	494	538	536	530	521	526	512	505	504	491	516	513	492	507	503	474	539	527	528	523	538	452	518	522	523	410	487	487	452	475	997		88.45	80.41	73.00	72.29	73.56	60.20	61.08	57.08	62.71
SbPEPR1	34	537	501	543	532	529	521	531	520	512	510	494	519	514	496	503	502	475	543	531	528	521	537	449	518	524	518	406	483	482	450	482	1003	996		82.67	72.66	72.18	74.20	61.42	62.53	58.52	64.27
SiPEPR1	35	532	502	545	531	523	518	522	513	505	512	493	521	518	509	507	501	477	541	534	533	534	536	451	533	537	529	414	495	492	452	482	912	907	930		73.84	73.71	75.11	60.32	61.01	56.66	62.38
BdPEPR1a	36	530	493	545	532	525	517	537	511	504	506	500	522	524	488	506	507	479	542	517	522	526	527	451	517	519	523	410	496	491	449	490	810	822	816	830		89.70	75.67	62.87	62.16	57.95	62.95
BdPEPR1b	37	530	497	549	534	530	525	536	507	505	504	497	521	520	488	505	505	479	536	518	522	521	527	453	519	523	526	416	495	494	449	488	805	814	812	824	1002		75.58	62.82	61.90	57.78	63.28
OsPEPR1a	38	550	504	547	545	539	530	547	517	507	508	502	528	527	500	512	511	483	535	526	523	517	539	453	527	529	530	417	500	500	460	480	817	829	834	845	843	845		63.17	62.51	57.60	63.84
OsPEPR1b	39	487	482	503	487	506	483	500	468	460	464	451	473	470	491	472	471	435	494	489	488	485	497	437	479	481	480	409	466	471	450	483	677	679	691	678	701	703	705		57.66	60.02	58.46
BdPEPR1c	40	525	463	525	519	508	503	509	500	496	499	485	495	489	479	488	498	459	510	504	506	503	519	431	494	490	497	387	482	487	445	466	703	700	716	698	708	705	712	655		82.32	76.17
BdPEPR2	41	486	458	493	477	487	482	491	464	461	465	449	455	455	468	458	469	422	491	472	477	470	487	405	462	460	464	393	452	458	440	458	659	657	673	651	663	661	659	650	917		70.21
HvPEPR1	42	536	496	532	519	525	511	526	510	505	510	499	500	509	489	502	505	460	531	524	526	517	523	443	499	496	506	397	479	482	444	474	705	708	725	703	705	710	715	653	863	799	

		1	2	3	4	5	6	7	8	9	10	11	12	13	14	15	16	17	18	19	20	21	22	23	24	25	26	27	28	29	30	31	32	33	34	35	36	37	38	39	40	41	42
PtPEPR1a	1		66.25	66.51	67.18	61.46	58.82	56.66	53.17	58.73	59.41	59.04	56.26	56.57	57.34	53.55	50.31	56.48	58.02	55.01	55.47	55.98	54.39	43.14	56.86	57.78	56.15	42.75	54.39	54.70	49.46	46.31	48.39	49.00	49.62	49.16	49.23	49.16	51.30	42.46	48.77	41.94	50.54
PtPEPR1b	2	428		54.78	54.80	55.36	53.92	59.45	52.86	50.64	48.61	49.23	49.52	47.99	47.99	43.59	45.43	49.61	51.62	45.52	45.68	46.85	47.22	42.38	48.22	48.07	47.07	47.20	47.25	47.25	50.60	48.45	41.60	42.06	42.09	42.24	42.06	42.77	42.99	41.42	39.08	40.81	42.06
LuPEPR1	3	431	355		63.43	58.33	57.10	54.48	49.00	54.55	54.62	56.02	53.86	55.25	55.86	51.00	46.07	53.16	56.79	52.77	53.38	52.99	50.92	42.70	55.08	55.85	54.53	40.46	51.15	51.61	46.70	45.71	48.93	50.31	50.15	49.69	49.46	49.08	50.61	42.33	47.78	41.81	49.08
CsPEPR1	4	434	354	411		62.54	60.37	59.44	53.17	57.50	58.18	58.42	55.80	57.50	57.34	53.24	48.92	54.48	58.02	55.62	55.93	57.06	53.16	44.99	56.24	56.39	55.38	41.36	52.70	53.00	47.30	48.46	49.31	49.77	50.54	50.54	51.38	51.76	51.45	45.23	49.08	43.01	50.38
PpPEPR1a	5	397	346	378	404		85.53	72.03	55.38	56.88	56.94	59.60	55.73	56.19	56.66	53.01	49.30	55.80	56.72	53.09	52.78	52.84	52.78	47.04	53.86	54.01	53.00	41.99	51.81	51.65	48.32	50.00	46.92	47.23	47.09	45.86	47.00	47.55	48.77	45.53	45.47	42.42	47.69
PpPEPR1b	6	380	337	370	390	532		69.94	54.90	55.49	55.40	58.67	55.11	55.73	56.66	50.70	48.22	54.56	56.11	52.78	52.16	52.53	51.70	47.04	51.70	51.54	51.16	39.74	50.87	51.18	47.52	49.36	46.31	47.08	46.78	46.01	47.16	47.70	48.01	44.09	45.47	42.58	47.23
MdPEPR1	7	366	343	353	384	448	435		58.18	57.51	54.78	56.81	56.85	53.56	54.49	49.77	50.24	55.92	54.87	51.39	51.08	51.77	50.00	47.25	51.62	50.70	51.70	46.10	50.16	51.10	50.17	55.82	44.84	45.61	46.08	45.16	47.92	47.38	48.54	44.43	44.77	44.52	46.69
VvPEPR1	8	344	305	318	344	345	342	338		52.47	49.77	50.23	50.24	48.07	49.46	44.29	45.15	47.79	52.09	48.30	48.30	48.08	46.45	53.93	44.60	45.52	45.37	50.47	46.31	45.99	48.34	56.23	40.52	41.14	41.17	40.86	41.14	41.08	42.37	49.10	40.00	41.94	41.91
CsaPEPR1a	9	380	317	354	372	368	359	360	329		81.64	55.71	55.71	52.78	53.55	50.23	48.64	56.01	53.31	49.69	50.15	51.30	49.69	44.57	51.77	52.08	51.69	41.44	48.38	48.84	42.68	47.53	45.31	46.54	46.40	45.94	47.16	46.78	47.38	41.08	44.48	39.63	46.24
CsaPEPR1b	10	385	315	355	377	369	359	355	323	529		56.70	52.70	54.55	55.01	52.46	46.62	54.92	53.54	50.84	51.46	52.14	50.54	42.86	51.38	51.38	51.61	38.92	48.62	49.38	43.85	45.55	46.01	47.09	46.64	46.48	47.32	46.64	47.55	41.17	45.64	40.80	46.93
GmPEPR1a	11	382	318	363	378	385	379	367	325	361	368		85.60	80.96	71.67	57.96	52.70	59.51	56.02	51.46	52.23	51.99	50.69	41.45	52.93	54.01	53.16	39.04	50.85	51.00	45.45	45.08	48.54	48.54	49.00	47.63	48.16	47.78	48.70	41.69	46.63	40.86	48.08
GmPEPR2	12	364	311	349	361	360	356	357	316	351	342	553		77.24	69.50	55.02	53.45	59.33	53.09	50.39	50.85	50.61	48.07	43.20	50.23	51.47	50.46	39.20	48.46	48.30	43.83	45.73	46.62	47.08	47.39	46.32	46.31	46.39	46.53	40.06	45.01	39.38	46.77
PvPEPR1b	13	366	310	358	372	363	360	346	311	342	354	523	499		70.59	56.57	51.62	59.51	54.63	51.16	52.08	51.07	50.54	41.60	50.77	51.85	51.77	37.50	49.00	48.69	44.07	44.46	47.93	47.93	48.39	48.55	48.01	47.78	47.63	41.08	45.40	40.40	47.16
MtPEPR1	14	371	310	362	371	366	366	352	320	347	357	463	449	456		55.64	51.00	58.58	55.56	52.08	51.77	50.92	48.84	42.06	50.62	51.54	51.16	37.35	47.92	48.07	43.45	45.85	47.16	47.31	46.86	47.47	47.55	47.17	47.78	40.92	44.94	39.02	46.70
PvPEPR1c	15	347	282	331	345	343	328	322	287	326	341	375	356	366	360		44.82	51.93	51.77	47.23	47.85	48.39	47.69	40.15	47.77	47.30	47.85	32.97	44.62	45.23	40.46	43.45	43.71	43.56	44.19	43.27	43.64	43.43	43.88	37.79	42.42	36.35	42.64
GmPEPR1b	16	326	283	299	317	319	312	313	279	304	303	341	333	334	330	290		68.86	47.22	44.46	45.23	44.87	43.36	42.36	43.03	43.19	42.75	36.61	40.06	39.91	34.82	41.28	40.00	40.46	40.49	39.94	41.08	41.01	41.76	34.59	37.63	31.54	39.08
PvPEPR1a	17	366	314	345	353	361	353	354	303	354	357	385	372	385	379	336	429		53.31	48.92	50.00	49.77	50.62	46.49	49.00	49.46	48.77	39.84	45.85	46.00	41.08	48.82	46.32	46.63	47.09	46.64	48.31	47.47	48.08	39.94	43.49	37.88	46.47
AcPEPR1	18	376	334	368	376	367	363	355	337	346	348	363	344	354	360	336	306	346		52.70	53.93	54.45	54.39	44.84	51.08	50.62	51.46	39.20	50.46	50.46	45.23	49.92	49.54	49.85	50.61	49.85	50.84	50.00	50.00	43.23	47.01	43.10	49.85
SlPEPR1	19	357	295	343	361	344	342	333	313	323	331	334	327	332	338	307	289	318	342		94.44	79.26	61.57	47.99	50.85	51.00	51.23	36.42	47.61	47.15	42.37	41.38	46.15	47.31	47.93	47.63	46.78	47.39	47.93	41.60	45.25	39.48	47.54
StPEPR1	20	360	296	347	363	342	338	331	313	326	335	339	330	338	336	311	294	325	350	611		80.80	61.57	47.38	51.16	51.62	52.00	37.04	48.38	47.61	42.53	41.69	46.77	47.93	48.39	47.78	47.39	48.01	48.09	41.91	45.71	40.09	48.15
NbPEPR1	21	365	305	346	372	344	342	337	313	335	341	339	330	333	332	316	293	325	355	516	526		60.68	47.85	50.69	50.23	50.38	36.25	47.39	47.70	42.33	42.57	47.25	48.47	48.62	49.24	49.39	48.78	48.78	42.27	46.33	40.43	48.32
InPEPR1	22	353	306	331	345	342	335	324	301	323	329	329	312	328	317	310	281	329	353	399	399	395		48.07	48.15	48.77	49.46	35.59	45.54	45.54	40.31	43.23	47.08	47.62	48.01	47.55	46.32	46.54	47.70	41.76	46.08	40.77	47.38
EgPEPR1	23	280	253	278	292	294	294	284	295	279	279	269	270	270	273	261	255	291	291	311	307	312	312		40.74	41.20	40.37	43.21	40.64	41.60	39.86	46.79	38.12	38.98	38.86	39.02	38.67	39.14	39.04	43.21	36.62	36.24	38.12
AlPEPR1	24	369	312	358	365	349	335	334	289	336	334	343	325	329	328	310	278	318	332	330	332	331	313	264		91.14	84.37	59.13	61.67	62.44	57.81	42.37	46.45	47.00	46.70	47.93	47.08	46.92	47.93	41.58	44.92	39.60	46.91
AtPEPR1	25	375	311	363	366	350	334	328	295	338	334	350	333	336	334	307	279	321	329	331	335	328	317	267	586		82.35	58.36	62.60	62.91	58.58	42.68	47.38	47.92	47.62	48.54	47.23	47.69	48.54	41.89	44.77	39.75	46.60
EsPEPR1	26	365	305	355	360	344	332	335	294	336	336	345	327	336	332	311	277	317	335	333	338	329	322	262	545	532		61.30	61.36	62.60	57.96	43.14	47.30	48.23	47.47	48.39	47.54	48.08	48.69	41.36	45.38	39.91	47.00
BrPEPR1	27	277	261	263	268	262	248	266	267	259	253	253	245	243	242	214	220	249	254	236	240	236	231	229	382	377	396		48.48	48.80	53.50	44.32	33.80	34.57	34.31	34.77	34.26	34.82	35.70	40.40	31.59	35.70	33.64
AlPEPR2	28	353	301	333	342	329	323	318	289	314	316	330	314	318	311	290	260	298	328	309	314	309	296	254	399	405	397	302		90.53	76.73	44.80	43.98	44.44	43.85	44.62	44.53	44.62	44.77	42.63	43.45	40.64	44.14
AtPEPR2	29	355	301	336	344	328	325	324	287	317	321	331	313	316	312	294	259	299	328	306	309	311	296	260	404	407	405	304	564		77.21	44.80	43.67	44.14	43.69	43.85	43.61	44.00	44.77	42.63	43.91	40.80	44.14
CrPEPR2	30	321	297	304	307	302	297	301	277	277	285	295	284	286	282	263	226	267	294	275	276	276	262	236	374	379	375	298	478	481		44.91	39.04	40.43	39.54	39.69	39.29	39.54	40.31	44.62	38.83	43.15	39.81
AcPEPR2	31	301	281	298	315	313	309	326	316	298	297	293	289	289	298	282	258	309	325	269	271	278	281	270	275	277	280	246	280	280	260		41.98	43.21	42.92	42.62	43.36	42.84	43.43	46.40	41.14	43.59	42.59
ZmPEPR1a	32	315	270	319	321	305	301	291	263	295	300	316	303	312	307	285	260	302	323	300	304	309	306	247	301	307	307	219	285	283	253	272		89.32	88.58	80.25	73.26	72.80	74.69	58.91	62.29	55.49	63.88
ZmPEPR1b	33	319	273	328	324	307	306	296	267	303	307	316	306	312	308	284	263	304	325	308	312	317	310	253	305	311	313	224	288	286	262	280	577		88.73	81.33	75.58	74.85	76.85	59.91	62.60	55.49	65.17
SbPEPR1	34	324	274	328	330	307	305	300	268	303	305	320	309	316	306	289	264	308	331	313	316	318	313	253	304	310	309	223	285	284	257	279	574	575		83.02	74.38	74.38	76.85	60.03	63.43	55.86	65.28
SiPEPR1	35	321	275	325	330	299	300	294	266	300	304	311	302	317	310	283	260	305	326	311	312	322	310	254	312	316	315	226	290	285	258	277	520	527	538		74.07	73.61	76.23	58.33	61.73	54.01	63.73
BdPEPR1a	36	321	273	323	335	306	307	311	267	307	309	314	301	313	310	285	267	315	332	305	309	323	302	251	306	307	309	222	289	283	255	281	474	489	482	480		87.94	78.36	60.90	62.19	55.40	64.14
BdPEPR1b	37	321	278	321	338	310	311	308	267	305	305	312	302	312	308	284	267	310	327	309	313	319	303	254	305	310	313	226	290	286	257	278	471	485	482	477	569		77.90	61.21	61.27	54.94	65.07
OsPEPR1a	38	335	279	331	336	318	313	315	275	308	311	318	302	311	312	287	271	313	327	313	314	319	311	253	312	316	317	231	291	291	262	281	484	498	498	494	507	504		62.35	64.51	55.86	67.13
OsPEPR1b	39	276	251	276	294	285	276	271	273	267	268	271	260	267	266	246	221	260	281	270	272	276	271	245	269	271	268	223	266	266	257	271	380	387	389	378	394	396	404		52.40	56.37	52.40
BdPEPR1c	40	318	254	312	320	296	296	291	260	290	298	304	293	296	293	277	245	284	307	295	298	303	300	238	292	291	295	205	282	285	252	267	403	405	411	400	403	397	418	339		76.57	74.19
BdPEPR2	41	273	242	273	280	266	267	268	247	258	266	266	256	263	254	237	205	247	281	257	261	264	265	220	257	258	259	206	254	255	249	255	359	359	362	350	359	356	362	332	477		63.68
HvPEPR1	42	329	273	320	328	310	307	303	272	301	306	313	304	307	304	278	254	303	325	309	313	316	308	247	304	302	305	218	286	286	258	276	412	421	423	413	415	421	435	338	480	412	

		1	2	3	4	5	6	7	8	9	10	11	12	13	14	15	16	17	18	19	20	21	22	23	24	25	26	27	28	29	30	31	32	33	34	35	36	37	38	39	40	41	42
GmPEPR1a	1		91.70	87.73	75.89	71.33	67.27	66.55	66.91	66.91	63.67	64.75	63.31	63.57	55.90	63.08	63.08	63.80	63.80	57.65	61.65	60.28	58.51	54.90	54.90	54.90	54.74	53.17	53.87	53.36	54.64	58.21	51.42	52.13	49.65	51.77	50.00	48.94	48.94	49.29	49.65	49.29	50.00
GmPEPR2	2	254		87.36	76.95	71.68	67.27	65.83	66.91	66.55	64.39	64.75	64.03	64.29	56.60	62.72	62.37	63.44	64.16	57.65	61.65	60.99	59.22	54.90	54.90	54.55	55.09	53.17	53.52	53.36	56.43	60.71	52.13	52.48	50.35	51.77	50.71	50.71	50.71	51.06	51.06	50.00	50.00
PvPEPR1b	3	243	242		76.60	70.61	66.19	65.83	66.55	66.91	62.95	64.39	63.31	63.21	56.25	60.93	60.57	62.37	63.44	58.01	60.22	61.70	59.22	54.55	54.90	53.85	55.09	53.87	54.23	54.06	54.64	56.43	50.35	51.06	48.94	49.65	48.94	48.94	48.94	51.06	50.35	50.35	50.00
MtPEPR1	4	214	217	216		67.25	62.90	61.35	60.99	62.77	60.64	59.93	59.57	59.15	52.23	55.48	55.12	56.89	58.66	54.04	59.72	56.49	56.45	52.07	52.41	52.76	53.63	52.43	52.43	51.92	51.58	54.74	48.42	48.07	47.02	47.72	49.12	48.77	48.77	49.12	48.42	48.77	48.42
GmPEPR1b	5	199	200	197	191		84.95	72.50	68.57	71.79	70.36	71.79	70.00	67.02	61.03	64.06	63.70	65.84	66.90	61.84	65.12	60.21	60.78	56.60	56.25	56.25	57.14	56.29	56.64	56.14	57.30	61.57	55.28	54.58	53.87	54.58	54.58	52.46	52.46	53.17	53.52	51.76	53.17
PvPEPR1a	6	187	187	184	178	237		70.97	67.03	69.53	69.18	67.38	68.46	65.48	59.17	63.21	62.86	64.29	65.71	62.06	65.00	58.66	59.01	55.75	56.10	56.79	56.64	55.79	55.79	54.93	56.79	60.71	54.42	54.06	53.00	53.36	53.71	53.00	53.36	53.00	53.36	52.65	54.77
PpPEPR1a	7	185	183	183	173	203	198		88.81	86.28	75.81	74.73	71.22	72.24	61.81	68.46	68.46	70.61	68.82	63.35	71.68	61.70	58.16	60.84	60.14	59.44	60.00	57.04	57.75	56.54	57.86	60.36	57.45	56.03	56.38	56.74	56.74	55.67	55.67	55.67	56.03	54.96	56.38
PpPEPR1b	8	186	186	185	172	192	187	246		82.31	70.40	70.76	67.63	69.04	59.03	66.31	66.31	67.74	67.03	62.28	68.46	60.64	58.51	56.99	56.99	55.59	56.49	54.93	55.63	55.48	53.21	56.79	56.74	56.03	55.32	55.67	55.32	52.84	53.19	53.90	54.26	52.84	54.26
MdPEPR1	9	186	185	185	177	201	194	239	228		74.73	73.65	70.50	69.40	60.07	66.31	66.31	67.74	66.67	63.35	68.82	61.70	60.28	60.49	60.14	59.79	58.95	58.45	59.15	57.95	58.21	60.71	57.09	56.38	56.03	56.74	54.96	53.55	53.55	53.90	54.61	53.90	55.32
CsPEPR1	10	177	179	175	171	197	193	210	195	207		73.65	73.74	70.11	61.81	68.10	68.10	69.18	67.38	64.77	65.95	59.57	58.51	60.49	60.14	60.14	61.05	58.45	59.15	58.30	62.14	61.43	56.38	54.61	56.03	56.03	54.96	53.90	53.90	53.90	54.61	53.19	55.67
PtPEPR1a	11	180	180	179	169	201	188	207	196	204	204		78.78	68.68	66.32	67.38	66.67	68.82	69.18	66.55	67.03	59.57	59.93	60.49	59.09	59.79	60.00	58.45	59.15	57.60	57.86	58.93	56.74	56.03	55.67	55.67	55.32	53.90	53.55	55.32	55.32	53.19	56.03
PtPEPR1b	12	176	178	176	168	196	191	198	188	196	205	219		67.97	62.98	65.00	64.29	65.36	67.14	64.18	67.50	59.72	57.60	58.89	58.19	58.19	58.04	56.84	57.54	57.39	61.57	61.92	57.60	55.48	55.83	56.18	55.83	53.36	52.65	53.71	53.36	54.77	57.60
VvPEPR1	13	178	180	177	168	189	184	203	194	195	197	193	191		58.76	67.97	66.90	69.40	69.75	62.06	65.12	62.32	55.99	55.21	55.56	54.86	55.75	54.20	54.55	54.90	58.30	59.01	58.30	57.60	55.63	57.24	56.18	55.48	55.83	54.42	54.42	53.00	56.18
LuPEPR1	14	161	163	162	152	177	171	178	170	173	178	191	182	171		62.15	61.46	60.42	59.03	59.52	60.42	56.25	53.12	58.97	58.62	59.31	58.62	57.29	57.29	56.79	56.60	56.94	53.47	52.43	52.78	52.78	53.82	53.47	53.82	54.17	53.47	51.04	54.86
SlPEPR1	15	176	175	170	157	180	177	191	185	185	190	188	182	191	179		98.19	89.17	79.42	70.71	67.15	59.01	56.38	58.04	58.04	57.69	57.89	55.99	56.34	55.32	57.65	57.65	56.18	55.12	56.54	56.89	55.12	54.42	54.42	55.48	56.89	54.77	57.95
StPEPR1	16	176	174	169	156	179	176	191	185	185	190	186	180	188	177	272		89.17	78.70	69.64	66.79	58.66	56.03	57.69	57.69	57.34	57.54	55.99	56.34	55.32	57.30	57.65	55.48	54.42	55.83	56.18	54.42	54.42	54.06	55.12	56.54	54.77	57.24
NbPEPR1	17	178	177	174	161	185	180	197	189	189	193	192	183	195	174	247	247		79.78	70.36	68.23	61.13	55.67	59.44	58.74	58.39	59.30	57.04	57.39	56.38	57.65	57.65	55.48	55.12	55.48	56.54	54.06	55.12	54.77	56.54	56.89	54.77	57.24
InPEPR1	18	178	179	177	166	188	184	192	187	186	188	193	188	196	170	220	218	221		68.21	68.59	57.60	55.32	61.19	61.54	61.54	61.05	58.80	59.15	58.16	56.58	56.58	59.01	58.30	59.01	58.30	58.30	57.95	57.95	57.24	58.30	54.42	58.66
EgPEPR1	19	162	162	163	154	175	175	178	175	178	182	187	181	175	172	198	195	197	191		63.21	57.54	54.04	55.59	55.94	56.64	56.64	53.87	54.58	53.17	53.71	54.06	55.63	55.28	54.93	55.99	54.23	55.99	55.63	55.63	54.93	55.28	55.99
AcPEPR1	20	172	172	168	169	183	182	200	191	192	184	187	189	183	174	186	185	189	190	177		63.96	55.32	60.14	59.79	60.14	61.75	55.63	55.63	54.96	57.30	59.43	58.30	56.54	58.30	57.95	56.18	56.18	55.83	55.83	56.18	54.77	57.60
AcPEPR2	21	170	172	174	161	171	166	174	171	174	168	168	169	177	162	167	166	173	163	164	181		58.66	52.94	52.60	51.90	52.43	52.96	53.66	52.45	54.09	56.58	52.31	51.96	51.25	52.67	51.25	51.60	51.96	52.67	53.02	51.60	53.38
PvPEPR1c	22	165	167	167	162	172	167	164	165	170	165	169	163	159	153	159	158	157	156	154	156	166		48.26	48.26	47.92	49.65	48.60	49.30	49.47	52.84	52.13	48.41	49.12	48.59	50.18	50.88	47.35	48.06	47.35	48.06	47.00	47.70
AlPEPR1	23	157	157	156	151	163	160	174	163	173	173	173	169	159	171	166	165	170	175	159	172	153	139		95.73	92.55	88.26	76.24	75.89	73.05	53.12	52.43	52.43	52.08	51.39	53.82	52.43	53.82	53.82	54.17	54.17	51.04	55.21
AtPEPR1	24	157	157	157	152	162	161	172	163	172	172	169	167	160	170	166	165	168	176	160	171	152	139	269		92.20	88.97	75.53	75.18	72.70	53.47	52.43	52.08	51.74	50.35	53.47	51.39	53.82	53.47	52.78	52.78	50.69	54.51
EsPEPR1	25	157	156	154	153	162	163	170	159	171	172	171	167	158	172	165	164	167	176	162	172	150	138	261	260		89.36	75.53	75.53	72.70	52.78	53.12	51.74	51.39	51.04	52.43	53.12	54.17	54.17	53.12	53.47	51.04	55.56
BrPEPR1	26	156	157	157	155	164	162	171	161	168	174	171	166	160	170	165	164	169	174	162	176	151	142	248	250	252		74.47	74.82	72.70	52.96	53.31	51.04	51.04	50.35	53.12	52.43	53.82	53.82	53.12	53.12	50.69	54.51
AlPEPR2	27	151	151	153	151	161	159	162	156	166	166	166	162	155	165	159	159	162	167	153	158	152	139	215	213	213	210		97.50	92.86	54.55	53.15	51.05	50.35	51.05	52.10	52.45	53.85	53.50	53.15	52.80	50.35	53.15
AtPEPR2	28	153	152	154	151	162	159	164	158	168	168	168	164	156	165	160	160	163	168	155	158	154	141	214	212	213	211	273		94.64	54.90	53.85	50.35	49.65	50.35	51.40	51.75	53.15	52.80	52.80	52.80	51.05	53.15
CrPEPR2	29	151	151	153	149	160	156	160	157	164	165	163	163	157	163	156	156	159	164	151	155	150	140	206	205	205	205	260	265		53.68	52.98	50.18	49.12	49.12	50.18	50.88	51.93	51.58	51.93	51.58	50.18	51.58
CsaPEPR1a	30	153	158	153	147	161	159	162	149	163	174	162	173	165	163	162	161	162	159	152	161	152	149	153	154	152	152	156	157	153		73.65	48.75	48.75	48.40	48.40	48.40	46.62	46.26	46.26	46.62	46.62	49.11
CsaPEPR1b	31	163	170	158	156	173	170	169	159	170	172	165	174	167	164	162	162	162	159	153	167	159	147	151	151	153	153	152	154	151	204		50.53	49.11	49.11	49.82	50.18	49.47	50.18	49.47	49.82	49.47	50.89
SbPEPR1	32	145	147	142	138	157	154	162	160	161	159	160	163	165	154	159	157	157	167	158	165	147	137	151	150	149	147	146	144	143	137	142		93.50	93.17	90.97	80.87	78.34	79.06	72.92	73.74	70.76	75.09
ZmPEPR1a	33	147	148	144	137	155	153	158	158	159	154	158	157	163	151	156	154	156	165	157	160	146	139	150	149	148	147	144	142	140	137	138	259		90.65	87.73	78.70	76.53	77.62	70.76	71.22	68.23	71.84
ZmPEPR1b	34	140	142	138	134	153	150	159	156	158	158	157	158	158	152	160	158	157	167	156	165	144	138	148	145	147	145	146	144	140	136	138	259	252		88.13	79.50	77.34	78.06	71.22	71.68	66.91	71.94
SiPEPR1	35	146	146	140	136	155	151	160	157	160	158	158	159	162	152	161	159	160	165	159	164	148	142	155	154	151	153	148	147	143	136	140	252	243	245		83.03	78.70	79.42	71.84	72.66	68.59	73.65
OsPEPR1a	36	141	143	138	140	155	152	160	156	155	155	156	158	159	155	156	154	153	165	154	159	144	144	151	148	153	151	150	148	145	136	141	224	218	221	230		79.78	80.87	70.40	71.22	66.43	71.84
BdPEPR1a	37	138	143	138	139	149	150	157	149	151	152	152	151	157	154	154	154	156	164	159	159	145	134	155	155	156	155	154	152	148	131	139	217	212	215	218	221		94.95	72.20	71.94	68.23	74.01
BdPEPR1b	38	138	143	138	139	149	151	157	150	151	152	151	149	158	155	154	153	155	164	158	159	146	136	155	154	156	155	153	151	147	130	141	219	215	217	220	224	263		73.29	73.02	68.23	75.09
BdPEPR1c	39	139	144	144	144	151	150	157	152	152	152	156	152	154	156	157	156	160	162	158	158	148	134	156	152	153	153	152	151	148	130	139	202	196	198	199	195	200	203		95.32	85.56	77.26
BdPEPR2	40	140	144	142	138	152	151	158	153	154	154	156	151	154	154	161	160	161	165	158	158	149	136	156	152	154	153	151	151	147	131	140	205	198	200	202	198	200	203	265		85.97	77.70
HvPEPR1	41	139	141	142	139	147	149	155	149	152	150	150	155	150	147	155	155	155	154	157	155	145	133	147	146	147	146	144	146	143	131	139	196	189	186	190	184	189	189	237	239		75.45
OsPEPR1b	42	141	141	141	138	151	155	159	153	156	157	158	163	159	158	164	162	162	166	159	163	150	135	159	157	160	157	152	152	147	138	143	208	199	200	204	199	205	208	214	216	209	

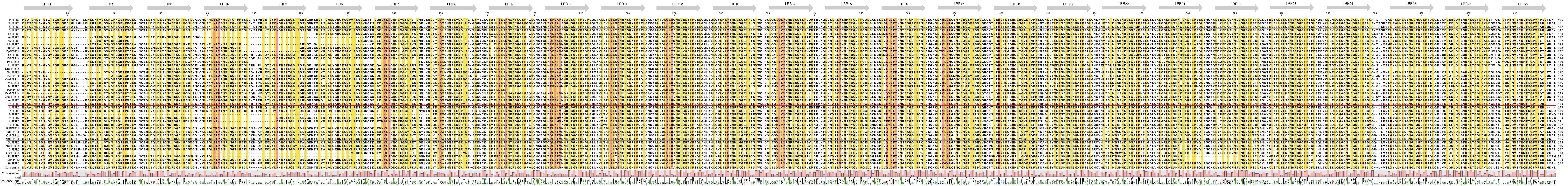
LRR1
LRR2
LRR3
LRR4
LRR5
LRR6
LRR7
LRR8
LRR9
LRR10
LRR11
LRR12
LRR13
LRR14
LRR15
LRR16
LRR17
LRR18
LRR19
LRR20
LRR21
LRR22
LRR23
LRR24
LRR25
LRR26
LRR27
Consensus
Conservation
Sequence logo
